# Pathways of ocean heat towards Pine Island and Thwaites grounding lines

**DOI:** 10.1038/s41598-019-53190-6

**Published:** 2019-11-22

**Authors:** Yoshihiro Nakayama, Georgy Manucharyan, Hong Zhang, Pierre Dutrieux, Hector S. Torres, Patrice Klein, Helene Seroussi, Michael Schodlok, Eric Rignot, Dimitris Menemenlis

**Affiliations:** 1 Jet Propulsion Laboratory, California Institute of Technology, 4800 Oak Grove Drive, Pasadena, CA USA; 2 Institute of Low Temperature Science, Hokkaido University, Sapporo, Japan; 3 California Institute of Technology, Pasadena, CA USA; 4 Lamont-Doherty Earth Observatory, Columbia University, NY, USA; 5 Laboratoire de Physique des Océans, IFREMER‐CNRS‐IRD‐UBO, Plouzané, France; 6 Earth System Science, University of California Irvine, CA, USA

**Keywords:** Physical oceanography, Cryospheric science

## Abstract

In the Amundsen Sea, modified Circumpolar Deep Water (mCDW) intrudes into ice shelf cavities, causing high ice shelf melting near the ice sheet grounding lines, accelerating ice flow, and controlling the pace of future Antarctic contributions to global sea level. The pathways of mCDW towards grounding lines are crucial as they directly control the heat reaching the ice. A realistic representation of mCDW circulation, however, remains challenging due to the sparsity of *in-situ* observations and the difficulty of ocean models to reproduce the available observations. In this study, we use an unprecedentedly high-resolution (200 m horizontal and 10 m vertical grid spacing) ocean model that resolves shelf-sea and sub-ice-shelf environments in qualitative agreement with existing observations during austral summer conditions. We demonstrate that the waters reaching the Pine Island and Thwaites grounding lines follow specific, topographically-constrained routes, all passing through a relatively small area located around 104°W and 74.3°S. The temporal and spatial variabilities of ice shelf melt rates are dominantly controlled by the sub-ice shelf ocean current. Our findings highlight the importance of accurate and high-resolution ocean bathymetry and subglacial topography for determining mCDW pathways and ice shelf melt rates.

## Introduction

In the Amundsen Sea, the Pine Island and Thwaites Glaciers are the two fastest-flowing outlet glaciers^1,2^, contributing to an ice loss equivalent of about 0.3 mm yr^−1^ of recent global sea level rise^3–6^. The bed topography of these glaciers slopes downward inland, to as deep as 2500 m below sea level, making the glaciers prone to marine ice sheet instability^7,8^ and thus further acceleration of grounded ice loss. These glaciers have been accelerating over the past two decades, hypothetically triggered by variable and relatively recent high basal melting of their ice shelves^9,10^.

The main cause for high basal melting of Pine Island Ice Shelf (PIIS) and Thwaites Ice Shelf (TIS) is the relatively warm modified Circumpolar Deep Water (mCDW, about 0.5–1.5 ºC, located below ~300–500 m depth)^11^. The mCDW flows via submarine glacial troughs onto the continental shelf break, travels a few hundred kilometers southward, and flows into the deeper and inner parts of ice shelf cavities, where it meets ice in the vicinity of grounding lines (see, e.g., refs. ^11–13^) forming a buoyant mixture of mCDW and glacial meltwater. Ice shelf melt rates near grounding lines (1) are generally a few orders of magnitude higher than at other locations of the same ice shelves (e.g., refs. ^14,15^) and (2) significantly impact future evolutions of glaciers and thus their contributions to sea level rise^8,16,17^. Thus, it is necessary to understand mCDW pathways as well as processes determining ice shelf melt rates near grounding lines. However, existing observations are sparse in time and space and the importance of cavity circulation has only been inferred for Pine Island Glacier in previous studies^9,12^. Existing ocean simulations with realistic configurations typically either aim at representing (1) temporal variability and use horizontal grid spacings of ~1–2 km or coarser (e.g., refs. ^18–26^), or (2) the spatial distribution of melt and use idealized geometries or forcing or finer horizontal grid resolutions in small model domains (e.g., refs. ^12,16,27^).

**Figure 1:**
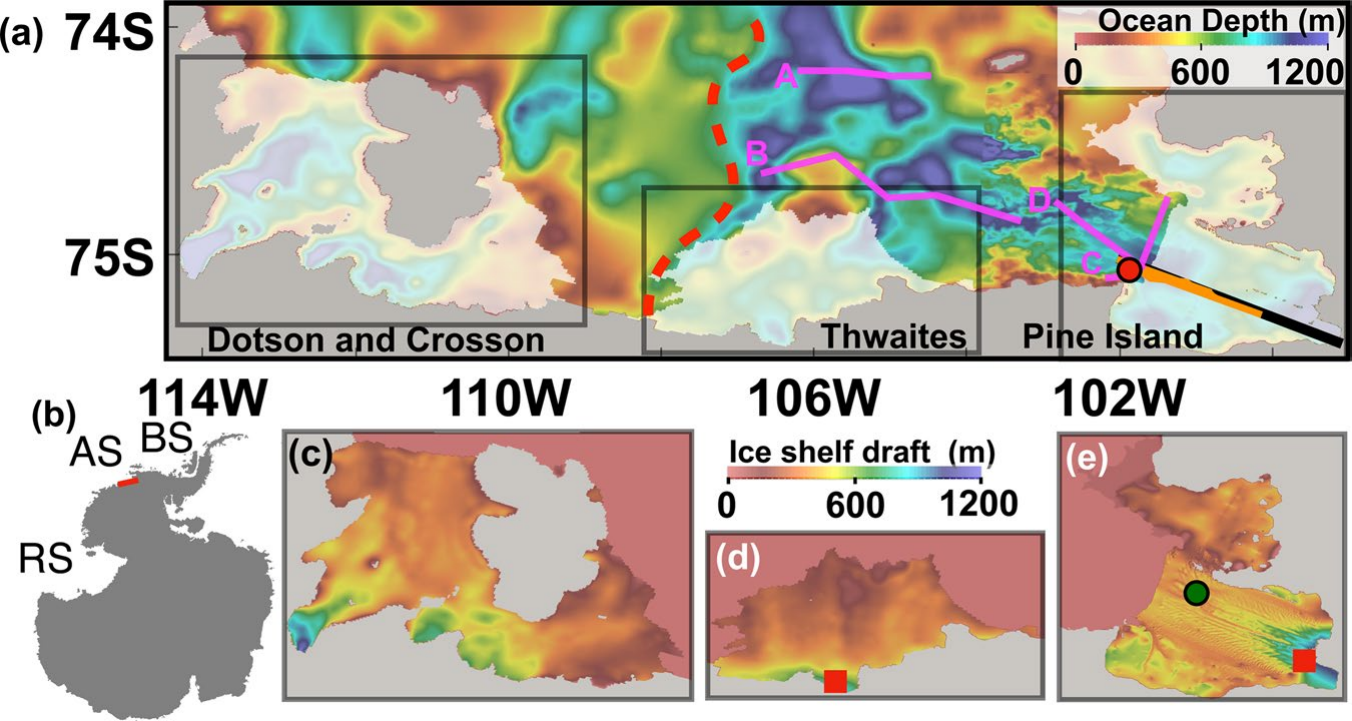
Model bathymetry and ice shelf draft. (**a**) Model bathymetry with partially-transparent white patches indicating locations of Dotson, Crosson, Thwaites, and Pine Island ice shelves. The red dot indicates the location of the Pine Island Ice Shelf (PIIS) front mooring. Underwater vehicle measurements are conducted in January 2009 along the orange line. The vertical sections of simulated ocean properties are shown along the black line. Red dashed line indicates topographic features separating deep and shallow regions. The lines marked A-D are sections discussed in the supplementary material. (**b**) The map of Antarctica with the region shown by red box denoting the model domain. AS, BS, and RS denote the Amundsen Sea, Bellingshausen Sea, and Ross Sea, respectively. The map is produced using Grid Analysis and Display System (GrADS 2.2.0, http://cola.gmu.edu/grads/grads.php). Ice shelf drafts for (**c**) Dotson and Crosson, (**d**) Thwaites, and (**e**) Pine Island ice shelves for the domains enclosed by gray lines in (**a**). Time series of ice shelf melt rates, thermal driving, and ocean speed below ice shelf are shown for the locations marked by the red squares and the green circle (Fig. 6 and Supplementary Fig. 21).

Here, we use a regional Eastern Amundsen Sea configuration (Fig. 1) of the Massachusetts Institute of Technology general circulation model (MITgcm) similar to refs. ^13,21^, but with horizontal and vertical grid spacings of 200 m and 10 m, respectively, and with some adjustments to model parameters (Supplementary Table 1) following refs. ^13,21,28^. We evaluate the results of this high-resolution configuration by comparing both its spatial and temporal characteristics to a variety of observations, including moorings in the ocean and various records of ice shelf melt. We aim to understand, for austral summer conditions, (1) pathways of warm mCDW from the continental shelf region (north of 74.24ºS) into the PIIS and TIS ice shelf cavities and towards their grounding lines and (2) processes that determine the magnitude of ice shelf melt near the PIIS and TIS grounding lines. For ice shelves, we assume a steady state for ice shelf thickness and cavity geometry and compute ice shelf melt rates following refs. ^29–31^. Although Dotson and Crosson ice shelves are in the model domain, they are located closer to the model boundary and we do not focus on these ice shelves for this study. We employ a passive tracer and Lagrangian particles (see Methods for details). We conduct a 60-day model simulation from 1 January 2010 to 1 March 2010, hereinafter CTRL after 30 days of spin-up. Sensitivity experiments with tides (TIDE) and without ice shelf melt flux (NOMELT) are also conducted (see Supplementary Table 2 and Methods for the details of surface forcing, initial and boundary conditions, and sub-ice shelf and ocean bathymetry).

## Observed and Simulated Ocean Properties in Ice Shelf Cavities and Melt Variability

**Figure 2:**
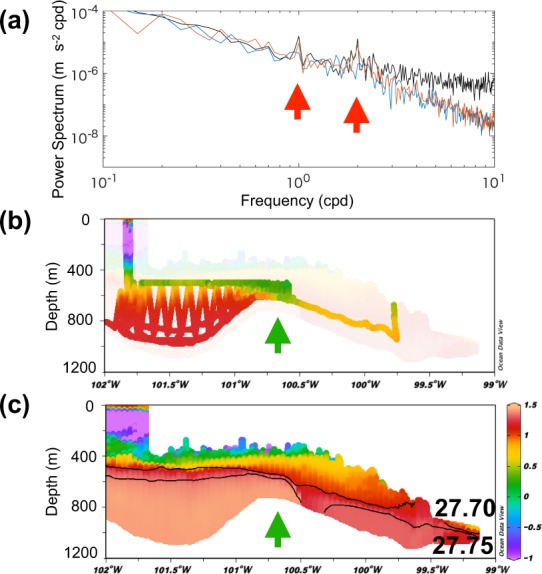
Comparison between model and observations. (**a**) The power spectra of ocean speed at 534 m depth from the BSR5 mooring data (black) and of simulated ocean speed for the CTRL (blue), and TIDE (red) cases. The diurnal and semi-diurnal tidal peaks are marked by red arrows. (**b**) Observed and (**c**) simulated vertical sections of potential temperature in the Pine Island ice shelf (PIIS) cavity. Isopyncal contours of 27.70 and 27.75 kg m^−3^ are shown with black lines. Underwater vehicle measurements are conducted in January 2009 along the orange line in Fig. 1a. The ridge separating the inner and outer parts of the PIIS cavity is marked by green arrows.

Existing observations are concentrated around the PIIS. Autosub3 Autonomous Underwater Vehicle (AUV) measurements under the PIIS cavity were carried out in 2009^32^ (along the orange line in Fig. 1a). They show that (1) a bathymetric ridge exists below the PIIS separating the ice shelf cavity into inner and outer parts (the green arrows in Fig. 2b,c)^32^ and (2) inflowing mCDW characterized by high temperature and high salinity is present in the outer part, while mCDW in the inner part is modified, being cooled and diluted (Fig. 2b and Supplementary Fig. 1). We note that observed hydrographic conditions (such as mCDW properties as well as thermocline depth) in the open water area near the PIIS were similar in 2009 and 2010^12,33^. The simulated vertical sections after 30 and 60 days of CTRL (along the black line in Fig. 1a) present features that are similar to the observations (Fig. 2 and Supplementary Figs. 1 and 2). Isopycnal contours of 27.70 and 27.75 kg m^−3^ are located at depths along which mCDW travels towards the PIIS grounding line. However, simulated mCDW properties are biased relative to obesrvations: the simulated mCDW located off the PIIS front is warmer and fresher by, respectively, ~0.3 °C and ~0.03.

The mooring observations located at the PIIS front (red circle in Fig. 1a) conducted between 2009 and 2014^34^ provide time series of ocean currents and potential temperature (See also Comparison with mooring observations in Supplementary text and Supplementary Fig. 3). At frequencies lower than about 2–3 cycles per day, the power spectrum of the observed currents at ~530 m depth matches well with the simulated currents. The addition of tides to the numerical model leads to a better representation of the diurnal and semi-diurnal spectral peaks (Fig. 2a). For frequencies higher than about 3 cycles per day, the model underestimates the energy spectrum by an order of magnitude. On the one hand, the model-data disagreement at high frequencies may be caused by the model’s inability to represent fast processes, such as higher-baroclinic-mode internal gravity waves, the variability of meltwater plumes, or the lack of remote internal gravity wave energy input at the northern model boundary. On the other hand, the data record could also have been affected by horizontal and vertical motions of the mooring, as the pressure sensor located at the same depth also contains similar short-timescale variability (Fig. 2c in ref. ^34^).

Conductivity-Temperature-Depth (CTD) observations were also obtained during 2010 in the Pine Island Bay region^33^. Overall, the simulated results capture the large-scale hydrographic structures (Supplementary Figs. 4–10). The main differences between model and observations are (1) simulated CDW properties ~0.3 ºC warmer than observations and (2) mismatch of thermocline depths and vertical structures, especially near the ice shelf fronts (see Comparison with CTD observations in Supplementary for detail).

**Figure 3:**
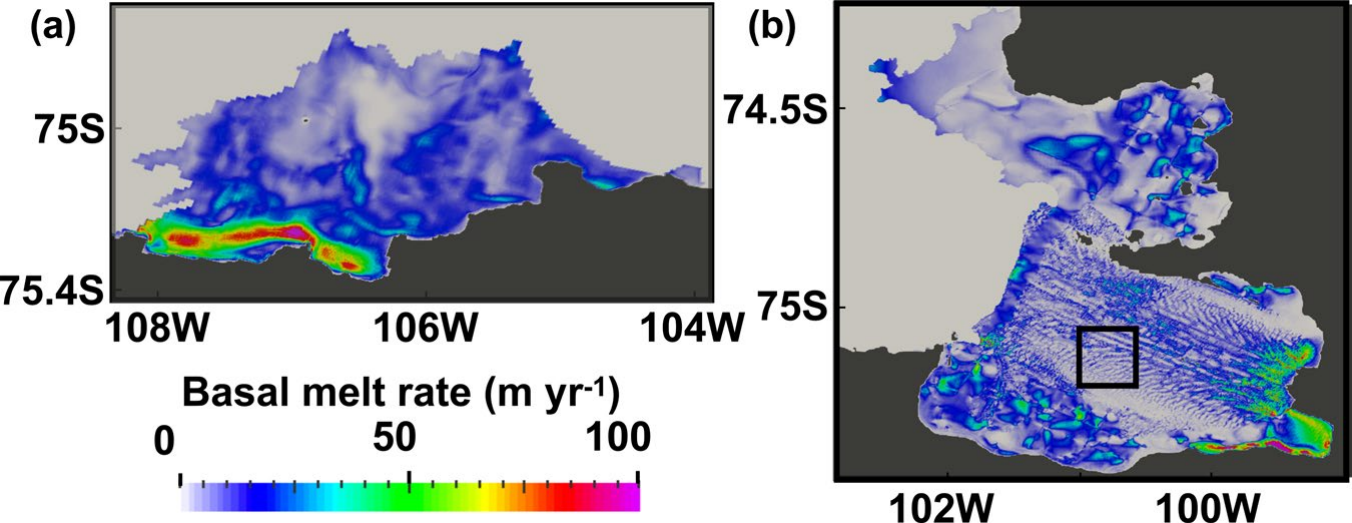
60-day mean simulated basal melt rates. 60-day-mean simulated basal melt rates for the (**a**) Thwaites ice shelf (TIS) and (**b**) PIIS. Close-up for the region enclosed by the black box in (**b**) is shown in Supplementary Fig. 19.

Satellite-based estimates^1^ of the integrated melt rates for the PIIS, TIS, and the Dotson and Crosson ice shelves are 101 ± 8 Gt yr^−1^, 97.5 ± 7 Gt yr^−1^, and 84 ± 18 Gt yr^−1^, respectively, based on data collected between 2003 and 2009. Simulated time series of integrated ice shelf melt rates for the PIIS, TIS, and the Dotson and Crosson ice shelves are 64.8 Gt yr^−1^, 65.1 Gt yr^−1^, and 83.7 Gt yr^−1^, respectively, and are nearly stable throughout the simulation period (Supplementary Fig. 11). The melt rates of PIIS and TIS are underestimated by ~30–40%. A recent study provided a high-resolution map of the PIIS melt rate between 2008 and 2015 using commercial sub-meter satellite stereo imagery and altimetry data, presenting melt rates of (1) 50–100 m yr^−1^ over the inner part of the cavity and (2) 10–30 m yr^−1^ over most of the outer part of the cavity (Fig. 3 and Figure 8 in ref. ^15^). Such features are well reproduced in our simulation. The observed ice shelf melt rates, however, peak near the grounding line at 100–200 m yr^−1^ while our simulated melt rates peak at 70–80 m yr^−1^ (Figs. 3 and S4 in ref. ^15^). These differences may be related to underestimated and poorly constrained heat and salt transfer coefficients^21^ and/or inaccurate ice shelf draft and ocean bottom bathymetry.

A year-long measurement of the PIIS basal melt rate near the ice shelf front (green dot in Fig. 1e) was conducted in 2014 using Autonomous phase-sensitive Radio-Echo Sounder (ApRES)^35^. Both observed and simulated time series of ice shelf melt rates at the same location appear to have fluctuations with frequencies of 7–10 days (see PIIS melt variability at ApRES location for details and arrows in Supplementary Fig. 12b), although the mean melt rates of the ApRES measurements are much lower, given that the thermocline depth was ~200m deeper in 2014^12^.

### Pathways of mCDW into pine island and thwaites grounding lines

**Figure 4:**
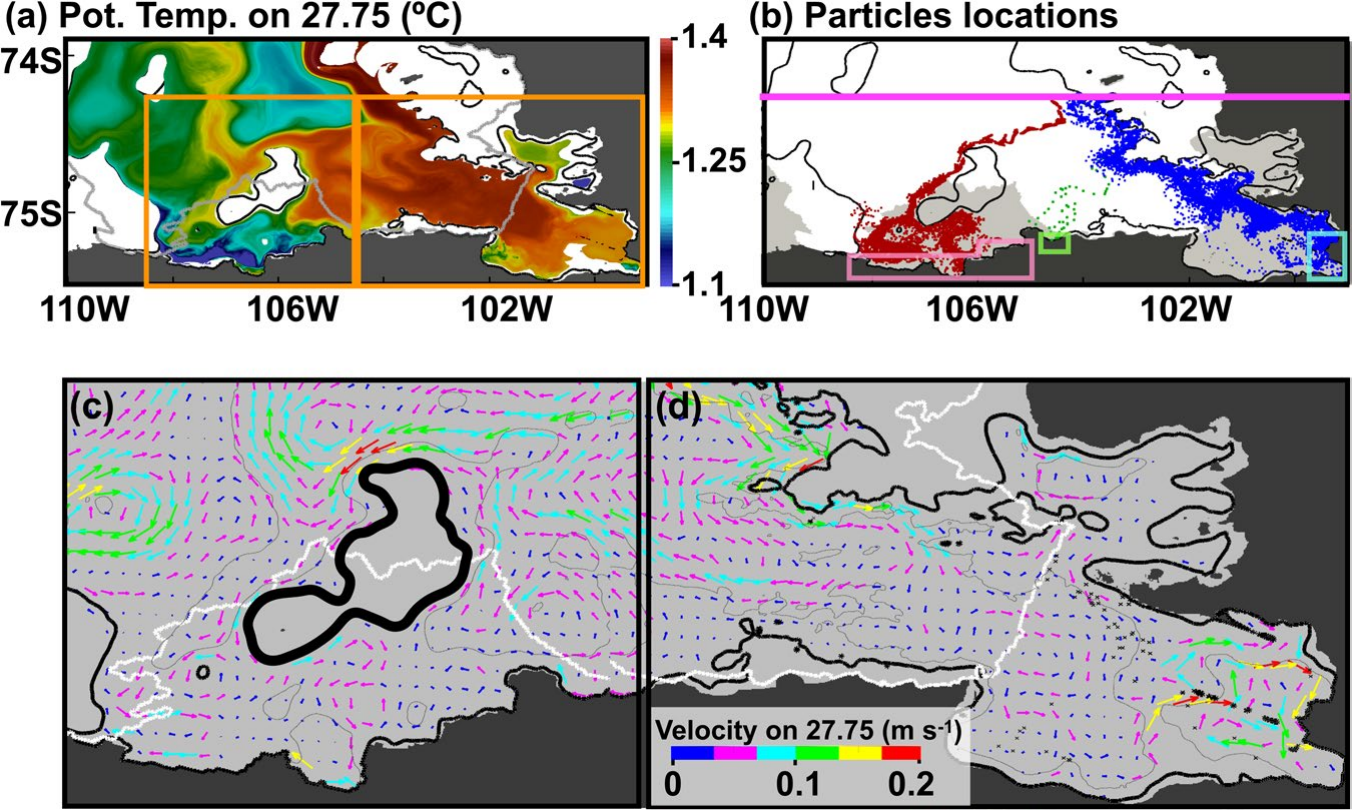
Pathways of mCDW into the PIIS and TIS cavities and towards their grounding lines. (**a**) Horizontal distribution of 60-day-mean potential temperature along 27.75 kg m^−3^ with two orange boxes denoting Pine Island and Thwaites regions. (**b**) Daily locations of all particle (initially released along 74.24° S (pink line)) before reaching the volumes in the vicinity of the PIIS and the eastern and western TIS grounding lines (highlighted by pink, light green, and cyan boxes) are shown by red, green, and blue points, respectively. Close-ups for (**c**) Thwaites and (**d**) Pine Island regions, enclosed by the orange lines in (**a**), showing 60-day-mean ocean current along 27.75 kg m^−3^ with directions (arrows) and speed (arrow color and arrow length). The thick black contour line in (**c**) indicates a bathymetric high. CDW flows around this contour, traveling southwards towards the middle part of the TIS grounding line. Locations of ice shelf fronts are shown with gray and white contours for (**a**) and (**c**,**d**) and location of ice shelve are shown with gray patches in (**b**).

Here, we show that mCDW pathways into the PIIS and TIS cavities and towards their grounding lines follow topographically constrained boundary currents using a passive tracer and particles (similar to refs. ^13,20,36,37^). mCDW reaching the PIIS grounding line has a potential density of 27.75 kg m^−3^ (Fig. 2c). Horizontal distribution of potential temperature on a 27.75 kg m^−3^ isopycnal shows that warm mCDW (~1.35 ºC) enters the model domain from the northern boundary and flows southward along bathymetric contours (Fig. 4a); some of this mCDW flows into the PIIS and TIS cavities (e.g., refs. ^18–21,38^). Sequential snapshots of tracer concentration representing mCDW (hereinafter mCDW tracer) released from the region north of 74.24ºS depict similar patterns to the potential temperature on the 27.75 kg m^−3^ isopycnal (Fig. 4a and Supplementary Fig. 13). Between 105–106.5ºW and 108–110ºW, the mCDW tracer travels southward with the southward flow (day 10, Supplementary Fig. 13a). The southward advection of mCDW tracer between 105–106.5ºW separates into multiple paths, some of which lead to the PIIS and TIS cavities (day 30) and travel towards their grounding lines after day 50–60 (Supplementary Fig. 13e,f). mCDW tracer also travels southward between 108–110ºW but it stays at the shallower depth (Fig. 1 and Supplementary Fig. 13) without mixing with main mCDW inflow towards the PIIS and TIS, likely because mCDW tracer between 108–110ºW is initially located on the shallower plateau separated by the ~600-m isobath (see red dashed line in Fig. 1a). We also note that particles released along 74.24ºS, advected based on hourly model outputs of velocity, also behave similarly to mCDW tracer (Supplementary Fig. 14).

**Figure 5:**
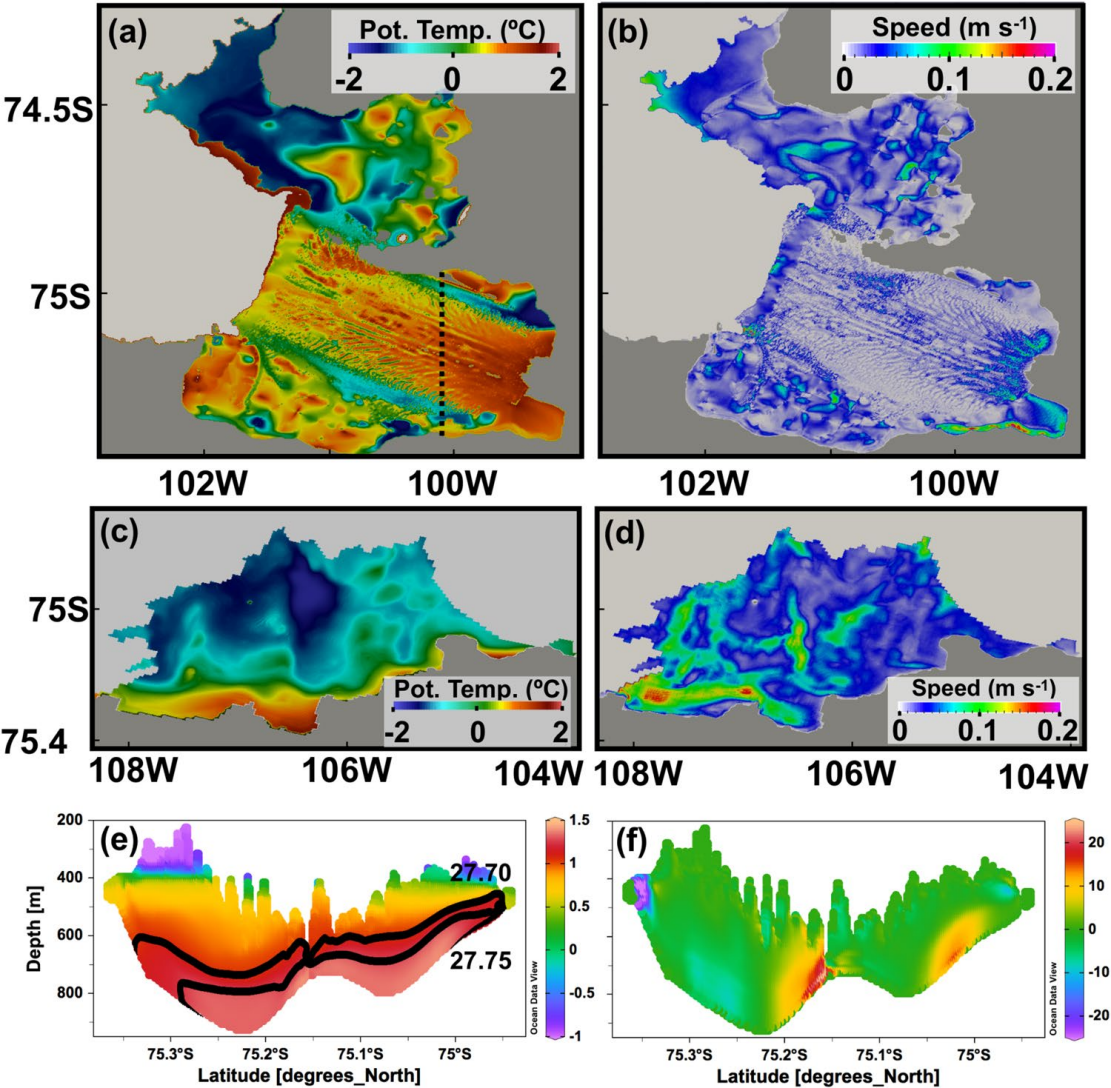
60-day-mean potential temperature and ocean speed in the PIIS and TIS cavities. Horizontal distributions of 60-day-mean (**a**,**c**) potential temperature and (**b**,**d**) ocean current at the uppermost model grid cell below the PIIS and TIS, respectively. Vertical sections of 60-day-mean (**e**) potential temperature and (**f**) ocean current along 100.1°W (dashed line in (**a**)).

We select all particles, which travel to the region near the PIIS and TIS grounding lines (pink, light green, and cyan boxes in Fig. 4b), and plot daily locations of these particles before reaching these boxes (red, green, and blue dots in Fig. 4b). We note that our analyses only show fast CDW pathways towards the PIIS and TIS grounding lines. Over a longer integration, one might find that more particles can reach the grounding zones of each ice shelf via more convoluted pathways. For the mCDW pathways towards the PIIS grounding line, mCDW flows southward along the 500-m bathymetric contour from the northeastern part of the model domain to the PIIS front (Fig. 4). Then, mCDW flows into the PIIS cavity and towards the grounding line following bathymetric contours (Fig. 4d). Under the PIIS, the mean sub-ice-shelf circulation is forced by ice shelf melting (Supplementary Fig. 18). Clockwise circulation with two cores of mCDW inflow towards the grounding line and strong outflow of mCDW-meltwater mixture following the southern edge is formed (Figs. 4c,d and 5e,f), with some similarities to previous model studies^12,16^.

Towards the TIS grounding line, mCDW takes different routes to the eastern and western parts. For the mCDW pathway towards the western part of the grounding line (red circles in Fig. 4b), where the main trunk of the Thwaites Glacier is located, mCDW passes through a similar region as for the PIIS but follows deeper isobaths (~1000 m) and flows westward separated from the main pathway to the PIIS cavity at the latitude of ~74.4ºS. Then, mCDW flows around the bathymetric high (thick black contour in Fig. 4c) and westward to 107ºW, where mCDW turns southward and flows into the TIS cavity and towards the grounding line. Warm mCDW is found along these pathways, although mCDW gradually cools as it approaches the grounding lines by mixing with surrounding water masses (Fig. 4a). Under the TIS, some portion of mCDW flows around the bathymetric high (thick black line in Fig. 4c) to the central part of the TIS and travels southwards towards the middle part of the TIS grounding line, whereas other portions of mCDW flow along the edge of the TIS ice front towards the western part of the TIS grounding line (Fig. 4b). For the mCDW pathways towards the eastern part of the TIS grounding line, mCDW passes through the same region, takes a similar route towards the PIIS, but flows westwards at the latitude of ~74.6ºS (green dots in Fig. 4b). On-site measurements are planned to be conducted on this part of the TIIS under the joint UK-US Thwaites projects.

All of these pathways towards the PIIS and TIS grounding lines have widths of 10–20 km (several baroclinic deformation radii). These pathways basically follow the 60-day-mean current on the 27.75-kg m^−3^ isopycnal (Fig. 4c,d) and thus bathymetric contours, which is confirmed by another particle release experiments advected by the 60-day-mean ocean current (see Particle Experiment in Supplementary text and Supplementary Figs. 15 and 16). Since bathymetry data are based on gravity data without *in-situ* measurements for the region off the western TIS front and beneath the TIS^39^, the CDW pathways towards the western TIS grounding remain uncertain.

Vertical sections of 60-day-mean potential temperature and ocean speed along 101.1ºW below the PIIS show (1) warm mCDW stored at depth (below 600 m) and Winter Water (WW) or modified mCDW located above and (2) two main inflows, which are topographically constrained boundary currents, transporting warm mCDW towards the PIIS grounding line (Fig. 5e,f). Inflows have their maximum speed close to the bottom. These topographically constrained boundary currents are critical for mCDW transport towards the PIIS grounding line. When ice shelf melting is excluded from the model, these sub-ice-shelf circulations stop completely but with little impact on open ocean circulation (Supplementary Figure 18)^23,38^.

We note that tidal forcing does not impact ice shelf melt rates for the PIIS and TIS^40^. Mean tidal currents based on tidal models^41,42^ are mostly small (<0.05 m s−1) over the Eastern Amundsen Sea continental shelf. Similar to a previous study^40^, the impact of tidal circulation on ice shelf melt rate is minor (Supplementary Fig. 11) and mCDW pathways remain similar for both the CTRL and TIDE cases (Supplementary Fig. 17).

### Mechanisms controlling ice shelf melt rates near grounding lines

Satellite observations^15^ show that the PIIS melt rate has small-scale patterns displaying (1) high basal melt rates (>100 m yr^−1^) associated with both basal keels and apexes of channels in the inner cavity near the grounding line, (2) high basal melt rate (20–40 m yr^−1^) associated with keels over the middle to outer part of the ice shelf, and (3) low basal melt rate (0–10 m yr^−1^) associated with channels over the middle to outer part of the ice shelf. These features are well reproduced in our model (Supplementary Fig. 19 and Figure 9 in ref. ^15^) but note that they cannot be produced by the spatial pattern of potential temperature. The simulated potential temperature of ocean water at the uppermost model grid cell below the ice shelf is ~0.5 ºC below channels and ~1.5 ºC below keels (Fig. 5). The thermal driving (the difference between *in-situ* temperature and freezing point) and thus ice shelf melt rates corresponding to this temperature difference fluctuates by only ~30%, which cannot explain the simulated spatial ice shelf melt pattern. Ocean velocity of the uppermost grid cell below the ice shelf, by way of contrast, shows small-scale features, consistent with previous studies^27,43^, that fluctuate from zero to as high as 0.3 m s^−1^ (Fig. 5), matching the spatial pattern of ice shelf melt rates.

For the TIS, high ice shelf melt rates are simulated near the grounding line but the spatial melt rate pattern is much smoother compared to that for the PIIS, possibly caused by smoothed ice shelf draft data from BEDMAP (Fig. 1). However, similarly to the case of the PIIS, the spatial pattern of ice shelf melt rate (Fig. 3a) matches that of ocean velocity of the uppermost grid cell below ice shelf (Fig. 5d).

**Figure 6:**
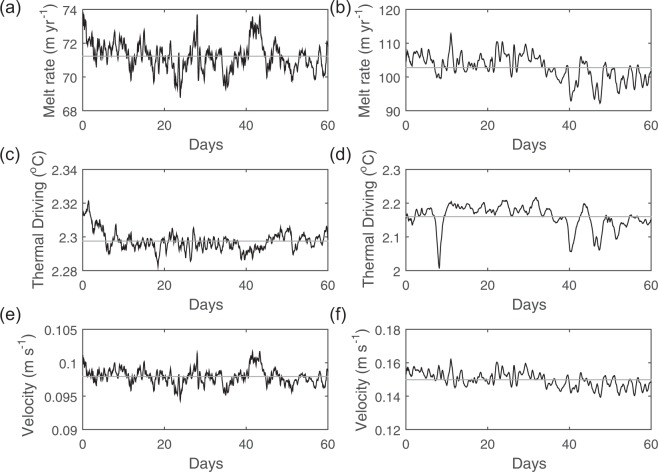
Time series of spatially averaged PIIS and TIS melt rate, thermal driving, and ocean speed near the grounding line. Time series of spatially averaged (**a**,**b**) ice shelf melt rates, (**c**,**d**) thermal driving and, (**e**,**f**) ocean speed below ice shelf for the location near the PIIS (left column) and TIS (right column) grounding lines, respectively, marked by red squares in Fig. 1d,e. Mean values are shown with gray lines. The spatial average is calculated for an area of 2 km by 2 km with its center located at 99.54°W, 75.34°S and 106.97°W, 75.26°S for PIIS and TIS, respectively.

The temporal variability of the PIIS and TIS melt rates are further investigated for the locations near the grounding lines and at the location of ApRES measurements (the red squares and the green dot in Fig. 1d,e, respectively). The spatially and temporally averaged PIIS and TIS melt rates (for the 2 km by 2 km area marked by red squares in Fig. 1d,e) are 71.3 Gt yr^−1^ and 102.6 Gt yr^−1^, respectively (Fig. 6). Temporal variabilities of ice shelf melt and ocean speed below ice shelf are similar, indicating that the observed variability of ice shelf melt rate at the locations near the PIIS and TIS grounding lines are primarily controlled by the fluctuation of ocean speed at the ice shelf base (Fig. 6). Correlation coefficients between basal melt rates and ocean speed below ice (thermal driving) are 0.97 (0.13) and 0.92 (0.73) for the PIIS and TIS, respectively, suggesting a strong control of ocean currents on ice shelf melt variabilities. For the TIS, the correlation coefficient between basal melt rate and thermal forcing is also high, suggesting some influence of thermal forcing on basal melt rate. Over the 60-day simulation period, time series of ocean current do not show obvious trends: they remain stable, being forced primarily by ice shelf melt at the location near the grounding lines. In contrast, at the location of ApRES measurements, short-term fluctuation of ocean speed causes the ice shelf melt to fluctuate with a frequency of 7–14 days (green dots in Fig. 1e), consistent with *in-situ* observations^35^ (See PIIS melt rate variability at ApRES location in Supplementary text). It is likely that both glacial-melt-driven and wind-driven circulations change the ice base velocity at the ApRES location (Supplementary Figs. 18, 20, and 21).

In this study, we conduct a regional eastern Amundsen Sea simulation with horizontal and vertical grid spacings of 200 m and 10 m, respectively. This simulation is conducted for 60 days from 1 January 2010 to 1 March 2010. The simulation is in qualitative agreement with existing observations and it suggests the existence of narrow, topographically-constrained pathways linking mCDW to the grounding lines of the PIIS and TIS. These pathways are relatively unaffected by fast ocean processes, showing little variability on timescales of days-months in our model simulation. mCDW passes through a small area around 104ºW and 74.3ºS, which therefore constitutes an ideal location for long-term *in-situ* monitoring. This also emphasizes the importance of accurate and high-resolution ocean and subglacial bathymetry for determining mCDW pathways and ice shelf melt rates. We also show that the temporal and spatial variability of ice shelf melt rates is primarily controlled by the sub-ice shelf ocean current. Identifying processes controlling ice shelf melt rate is an important step towards better projections of Antarctic mass balance and thus future contributions from the Antarctic ice sheet to sea level rise.

## Methods

We use a regional configuration of the Massachusetts Institute of Technology general circulation model (MITgcm) with hydrostatic approximation, dynamic/thermodynamic sea-ice^44^, and thermodynamic ice shelf^45^. The model domain contains the continental shelf region of the eastern Amundsen Sea and the Pine Island, Thwaites, Crosson, and Dotson ice shelves (Fig. 1). Nominal horizontal and vertical grid spacings are 200 m and 10 m, respectively. Model bathymetry is based on the International Bathymetric Chart of the Southern Ocean (IBCSO^46^), with recent updates of more accurate bathymetry for the region near Pine Island, and the Crosson and Dotson ice shelves^12,39^. The trough extending towards the Pine Island Ice Shelf is well mapped^39^ and we do not find obvious differences among different bathymetry datasets. The simulated ice draft was obtained using high-resolution observations from commercial, sub-meter satellite stereo imagery for the PIIS^15^ and Antarctic Bedrock Mapping (BEDMAP-2^47^) for the Thwaites, Dotson, and Cosson ice shelves. For ice shelves, we assume steady-state ice shelf thickness and cavity geometry and compute ice shelf melt rates following refs. ^28–30,45^.

Similar to refs. ^13,21^, atmospheric forcing is provided by the ongoing ECCO LLC270 optimization^48^, which is based on ERA-Interim^49^ and has been adjusted using the ECCO adjoint-model-based methodology^50^. Lateral ocean boundary conditions are derived from an extended (2001–2016) ocean simulation based on ref. ^13^, with increased (70) vertical levels. Initial conditions are derived from a 30-day spin-up from an initial rest state with potential temperature and salinity from the January 2010 monthly-mean fields from the extended simulation based on ref. ^13^. There is no additional freshwater runoff, that is, all calving icebergs are assumed to be transported and melt outside the regional domain. We start our simulation without sea ice and sea ice does not form until the end of the model simulation.

For the passive tracer representing mCDW, initial tracer concentrations are set to 1.0 for the region north of 74.24°S with potential temperature higher than 1.0°C, similar to ref. ^13^, and no tracer restoring is applied. Particle release experiments are conducted offline using hourly outputs of ocean current using Octopus (https://github.com/jinbow/Octopus) and particles are released along 74.24°S at the depth of the 27.75 kg m^−3^ isopycnal.

Two other sensitivity experiments are also conducted. For the TIDE case, tidal currents from the CATS2008A inverse barotropic tide model^41,42^ are superimposed on the ocean lateral boundary condition. For the NOMELT case, heat and salt transfer coefficients at the interface between ice shelf and ocean are set to zero for all ice shelves in the model domain. The comparison of model and data [e.g.,^9–15,31,32,34,35,51^] is shown in the main text and supplementary. Some of the figures are created with software Paraview and Ocean Data View^52,53^.

## Supplementary information

Supplementary info

## Data Availability

The model code and daily outputs are available at https://ecco.jpl.nasa.gov/drive/files/ECCO2/High_res_PIG. Each user must first register for an Earthdata account at https://urs.earthdata.nasa.gov/users/new in order to access these files.
