# Supplementary material for: Pathways of ocean heat towards Pine Island and Thwaites grounding lines
Source: Sci Rep. 2019 Nov 22;9:16649. doi: 10.1038/s41598-019-53190-6 (PMC6874652; doi:10.1038/s41598-019-53190-6)
Supplement: Supplementary file 1 — Supplementary info [file 41598_2019_53190_MOESM1_ESM.pdf]

Supplementary Information

# Pathways of ocean heat towards Pine Island and Thwaites grounding lines.

Yoshihiro Nakayama[1,2]*, Georgy Manucharyan[3], Hong Zhang[1], Pierre Dutrieux[4], Hector S. Torres[1], Patrice Klein[1,5], Helene Seroussi[1], Michael Schodlok[1], Eric Rignot[1,6], Dimitris Menemenlis[1]

**Affiliations:**

[1] Jet Propulsion Laboratory, California Institute of Technology, 4800 Oak Grove Drive, Pasadena, CA, USA

[2] Institute of Low Temperature Science, Hokkaido University, Sapporo, Japan

[3] California Institute of Technology, Pasadena, CA, USA

[4] Lamont-Doherty Earth Observatory, Columbia University, NY, USA

[5] Laboratoire de Physique des Océans, IFREMER - CNRS - IRD - UBO, Plouzané, France

[6] Earth System Science, University of California Irvine, CA, USA

*Correspondence to: Yoshihiro Nakayama (Yoshihiro.Nakayama@lowtem.hokudai.ac.jp)

## Comparison with CTD observations

Conductivity-Temperature-Depth (CTD) observations were obtained in 2010 in the Pine Island Bay region[33]. The simulated and observed vertical profiles of potential temperature and salinity for sections A-C capture large-scale hydrographic structures, showing WW with temperature minimum at 100-300 m and warm CDW below (Supplementary Figures 4-10). For section A, CDW properties at the depth of 500 m are warmer and fresher by ~0.3ºC and 0.04, respectively (Supplementary Figures 4 and 5 b-c, e-f). For section B, simulated CDW properties are warmer than observations, which is likely caused by too warm CDW properties at the northern model boundary (Figure 2 and Supplementary Figure 6-7). A previous study[51] noted the existence of a gyre in the Pine Island Bay based on hydrographic data collected in 2009. However, we do not detect significant gyre circulation in our simulation (Supplementary Figure 10). For section C, simulated CDW properties are warmer than the observations, which is likely caused by too warm CDW properties at the northern model boundary (Supplementary Figure 8-9). The simulated thermocline depth along section C is shallower than the observations by 100-200 m.

## Comparison with mooring observations

Observed time series of potential temperature do not show large fluctuations and water mass properties become more stable in the deeper part of the water column (Supplementary Figure 10). Such features are well reproduced in the CTRL case. We note that the simulated time series of potential temperature show warm bias for all depths, which is likely caused by (1) simulated CDW properties ~0.3 ºC warmer than observations (discussed above) and (2) simulated thermocline depth ~100 m shallower than observations (Figures 2b-c).

## Particle experiments

To test the importance of mean ocean current, we perform another particle release experiment using 60-day mean ocean currents. Paths and timescales of CDW transport into the PIIS cavity do not change appreciably (Supplementary Figure 15), confirming the importance of the ocean mean current (Supplementary Figure 15). We note, however, that no particles are found in the TIS cavity for the particle release experiment that uses the 60-day mean ocean currents. This is because the particles that are supposed to travel southwards to the TIS flow northwards instead (see red and blue arrows in Supplementary Figure 16) for this particle release experiment. This does not mean that the mean current plays an unimportant role in determining CDW pathways into the TIS. Instead, this emphasizes the importance of ocean eddies and short-time variability to help bridge the gap between mean ocean current systems. Indeed, particle pathways into the TIS (red dots in Figure 4b) follow ocean mean current on the 27.75 kg $m^{-3}$ isopycnal (Figure 4d) reasonably well.

## PIIS melt variability at ApRES location

A year-long measurement of the PIIS basal melt rate near the ice shelf front (green dot in Figure 1e) was conducted in 2014 using Autonomous phase-sensitive Radio-Echo Sounder (ApRES)[35]. Here, we compare their summer measurements (between January 14 and March 14, 2014) with simulated time series at the nearest model grid (Supplementary Figure 12). Since the hydrographic conditions in 2014 are colder than those in 2010 due to the deeper thermocline[35], the observed basal melt rates are lower than the simulated rates (Supplementary Figure 12a).

However, the observed and both the CTRL and TIDE simulated time series appear to have fluctuation with frequencies of 7–10 days (see arrows in Supplementary Figure 12b).

A previous study hypothesized that this low-frequency variability is caused by vertical displacement of the thermocline, forced by atmospheric forcing. In our model simulation, however, low-frequency variability is only present in ocean speed but not in potential temperature (Supplementary Figure 20). This indicates that bottom ocean currents play a dominant role in controlling short-timescale ice shelf melt variability. Such short-term ocean variability originates from the open ocean. Horizontal distributions of ocean speed anomaly 50 m below the ocean top cell (or ice shelf base) for days 14–17 (Supplementary Figure 21) show that the eddy with anticlockwise circulation approaches closer to the ice shelf front (days 14 and 15) and higher ocean speed signal propagates inward from open ocean area into the PIIS cavity (days 16 and 17), for example.

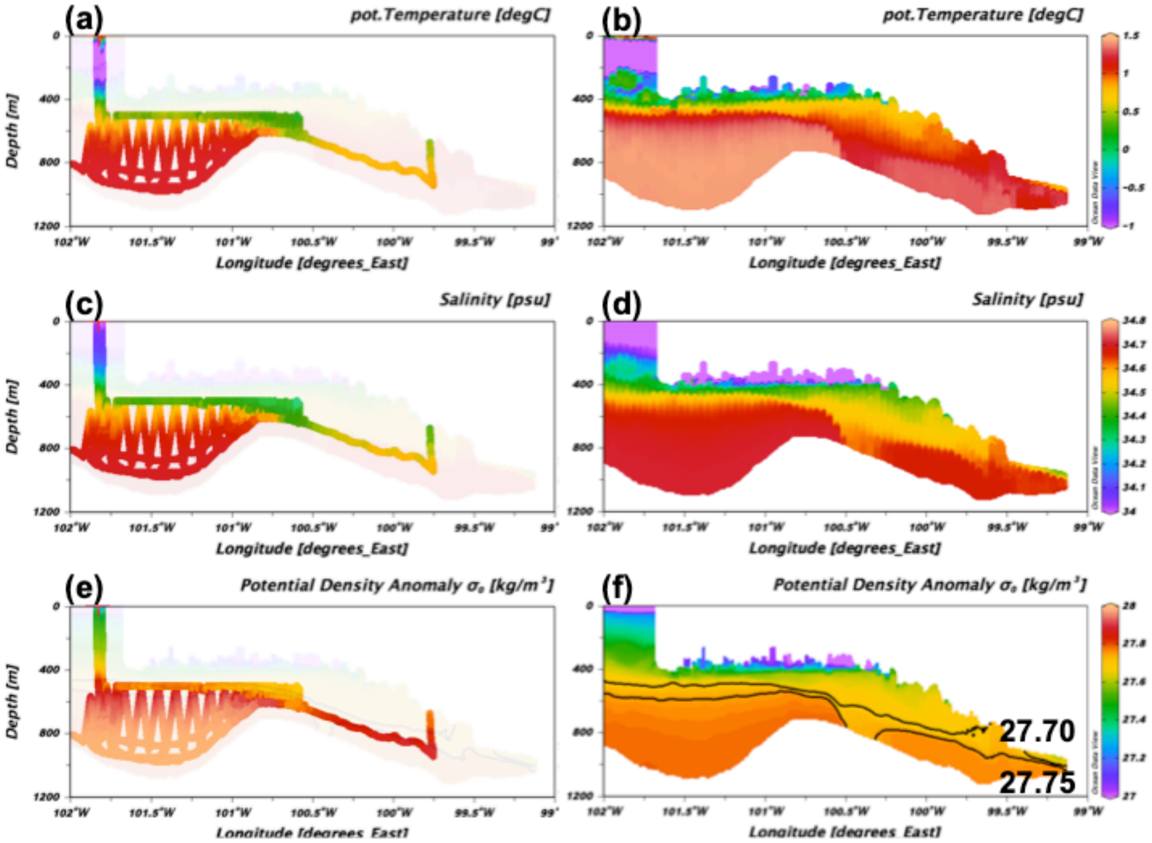


**Supplementary Figure 1.** Observed vertical sections of (a) potential temperature, (c) salinity, and (e) potential density in the Pine Island ice shelf (PIIS) cavity for January 2009. Underwater vehicle measurements are conducted along the orange line in Figure 1. Simulated vertical sections of (b) potential temperature, (d) salinity, and (f) potential density in the PIIS cavity for day 30 along the black line in Figure 1.

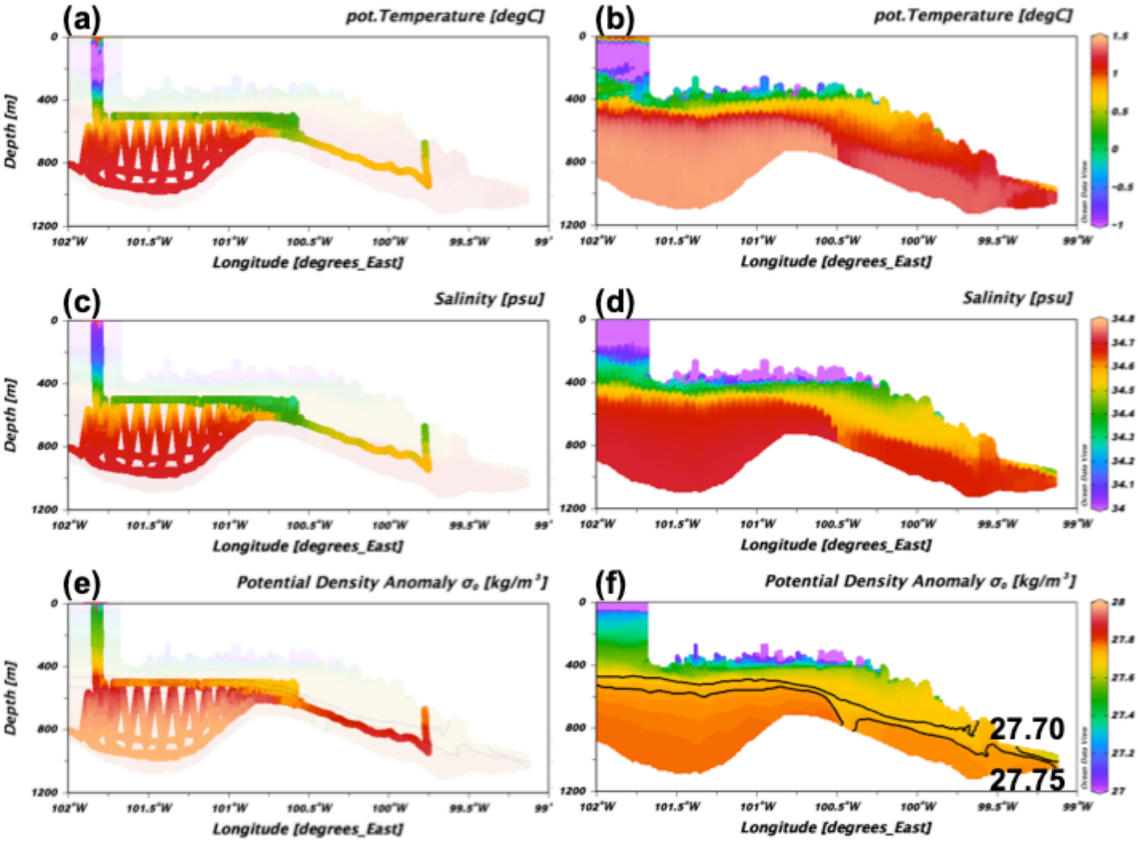


**Supplementary Figure 2.** Same as Supplementary Figure 1 but for simulated vertical sections for day 60.

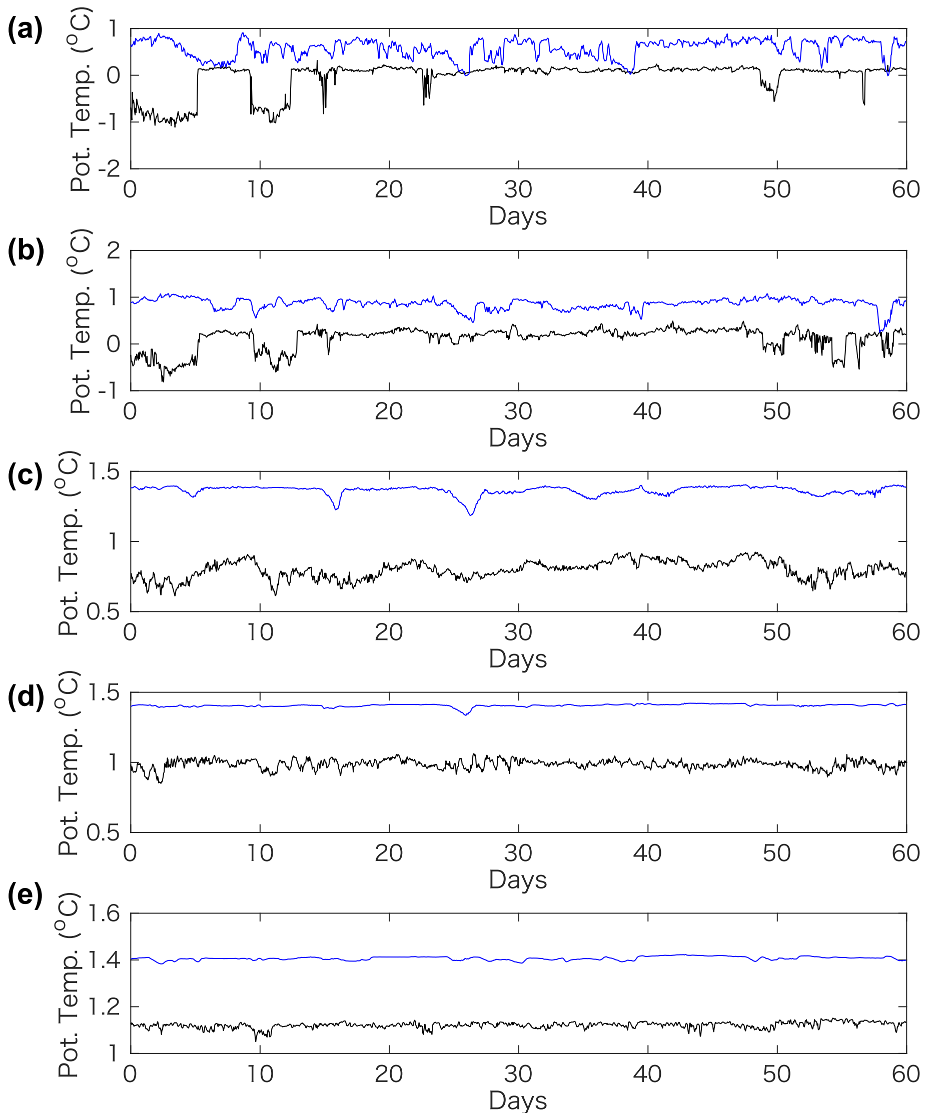


**Supplementary Figure 3.** The time series of potential temperature from the BSR5 mooring data (black) at the mean depths of (a) 783 m, (b) 698m, (c) 611 m, (d) 482 m, and (e) 429 m. The time series of simulated potential temperature at the depths of (a) 780 m, (b) 700 m, (c) 610 m, (d) 480 m, and (e) 430 m for the CTRL case (blue). Since no salinity data is obtained in the mooring measurements, salinity of 34.75 is assumed to convert from in-situ to potential temperature. These observations are obtained at the same time as the model simulation.

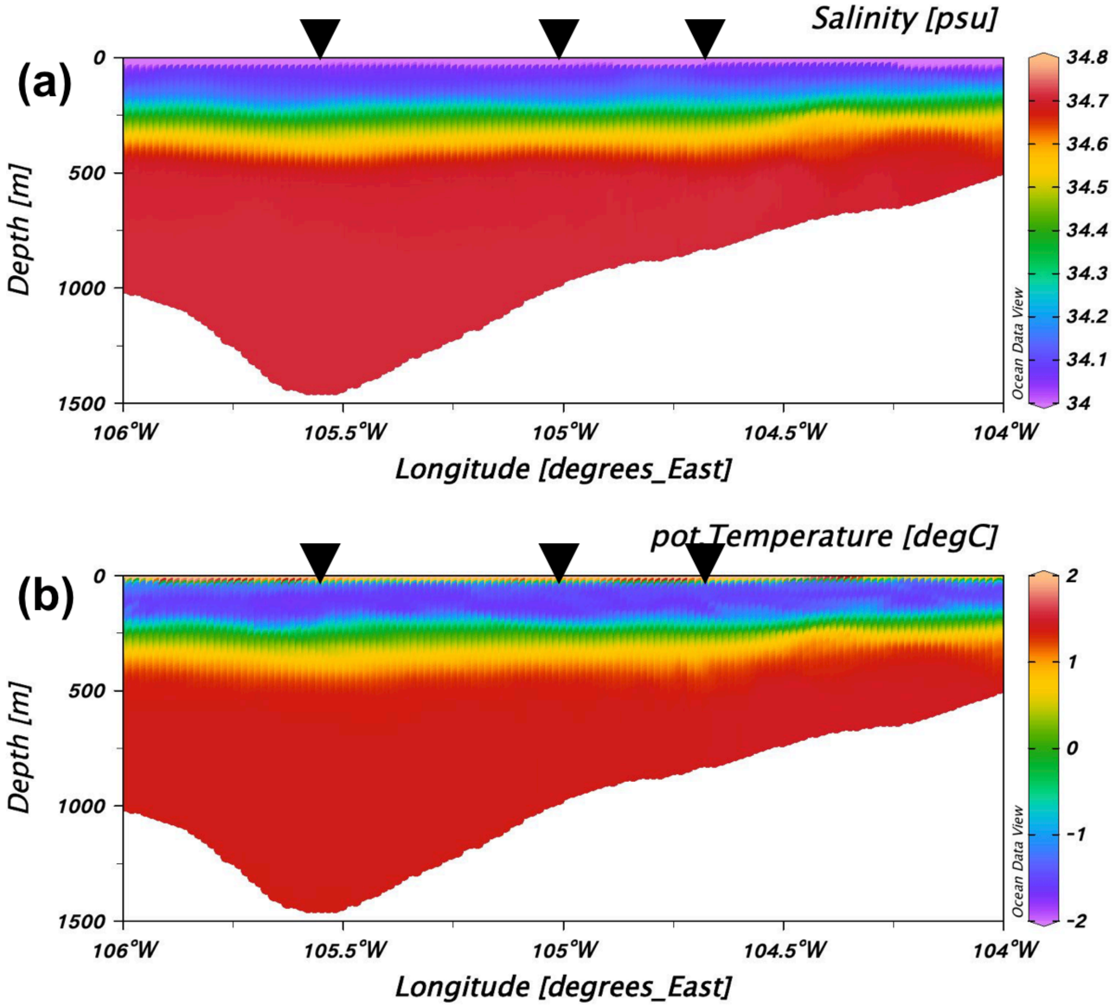


**Supplementary Figure 4.** Simulated vertical sections of (a) salinity and (b) potential temperature on January 30, 2010 along section A in Figure 1. Black triangles indicate the locations of CTD observations.

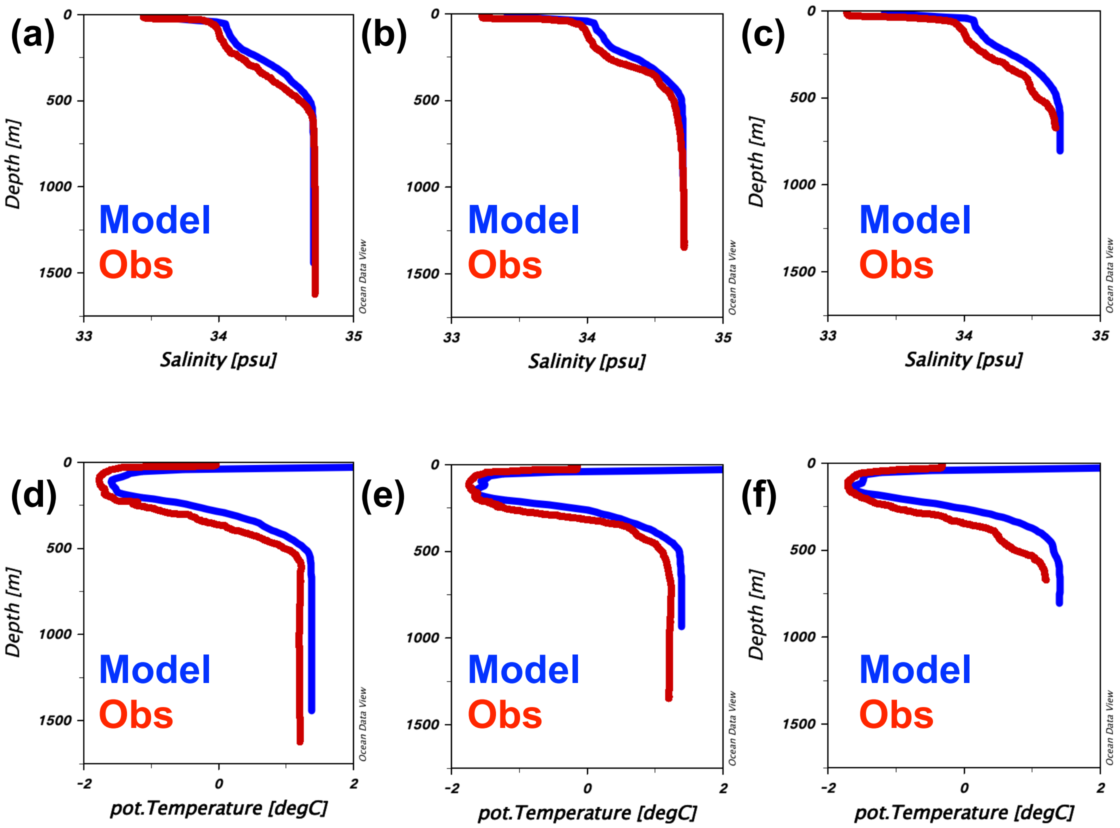


**Supplementary Figure 5.** Observed and simulated vertical profiles of salinity and potential temperature at the locations indicated by the black triangles along section A in Supplementary Figure 4 (ordered from left to right).

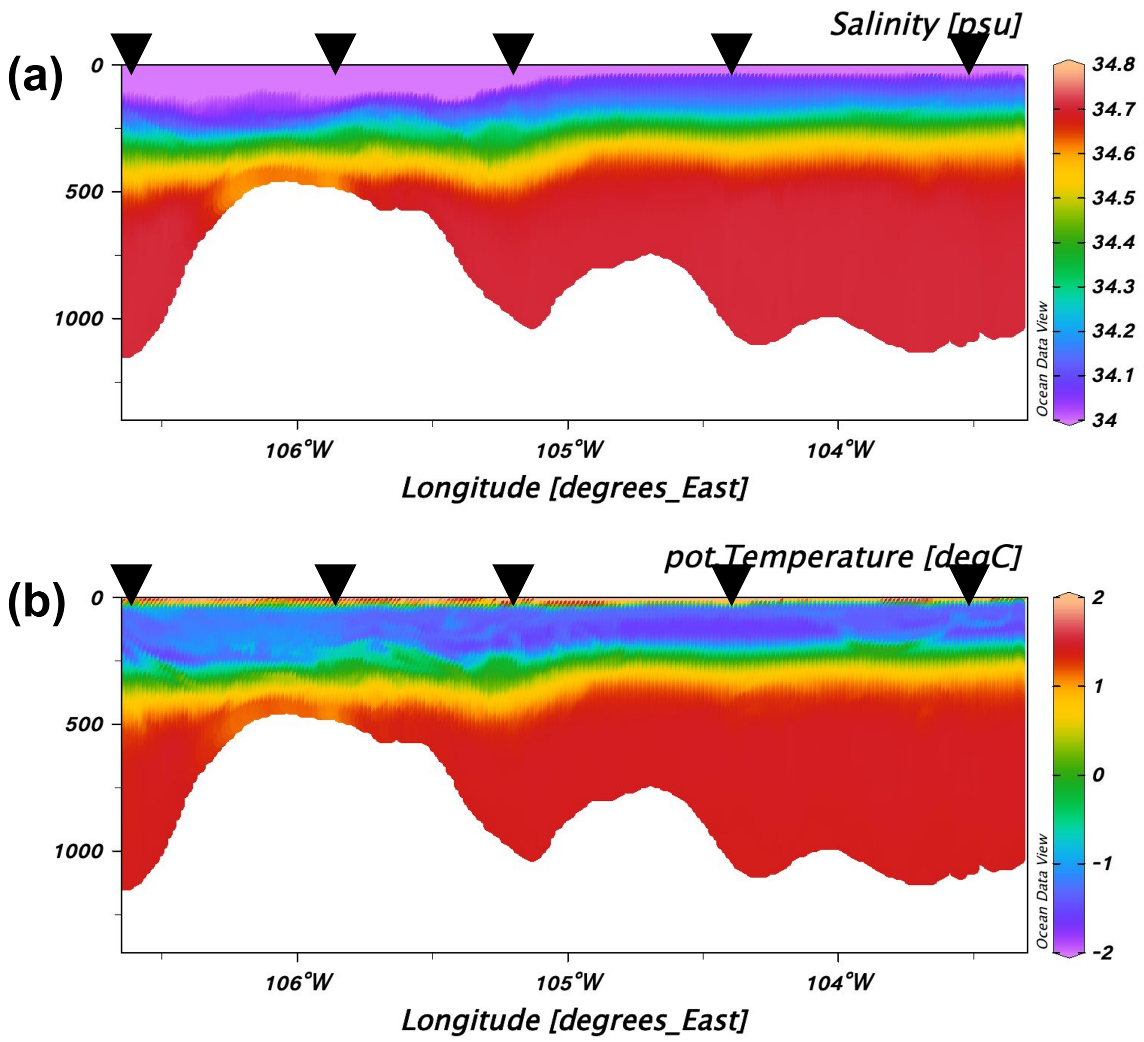


**Supplementary Figure 6.** Simulated vertical sections of (a) salinity and (b) potential temperature on January 30, 2010 along section B in Figure 1. Black triangles indicate the locations of CTD observations.

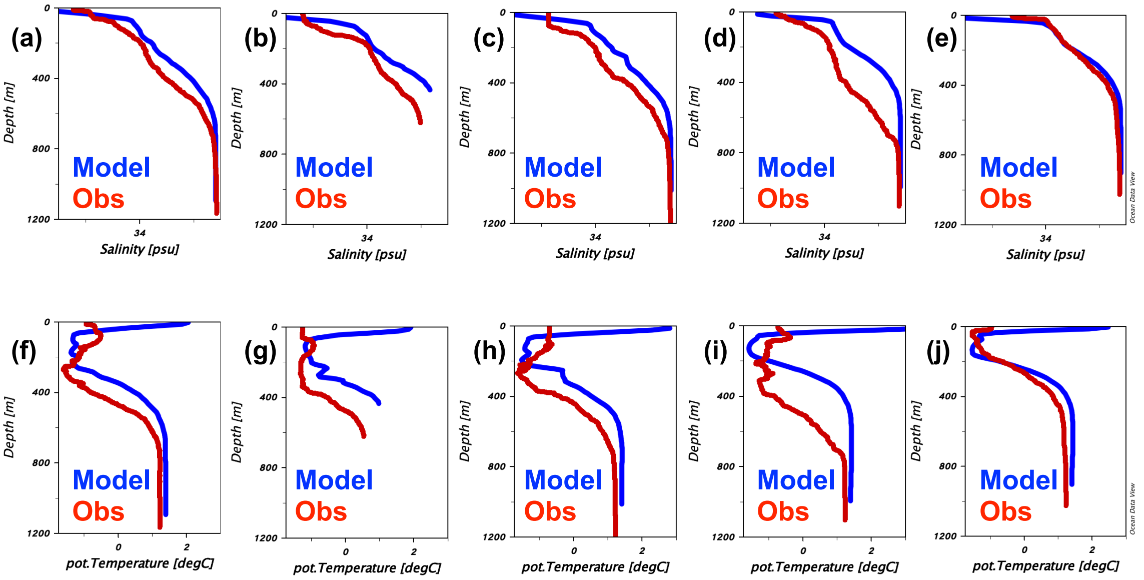


**Supplementary Figure 7.** Observed and simulated vertical profiles of potential temperature and salinity at the locations indicated by the black triangles along section B in Supplementary Figure 6 (ordered from left to right).

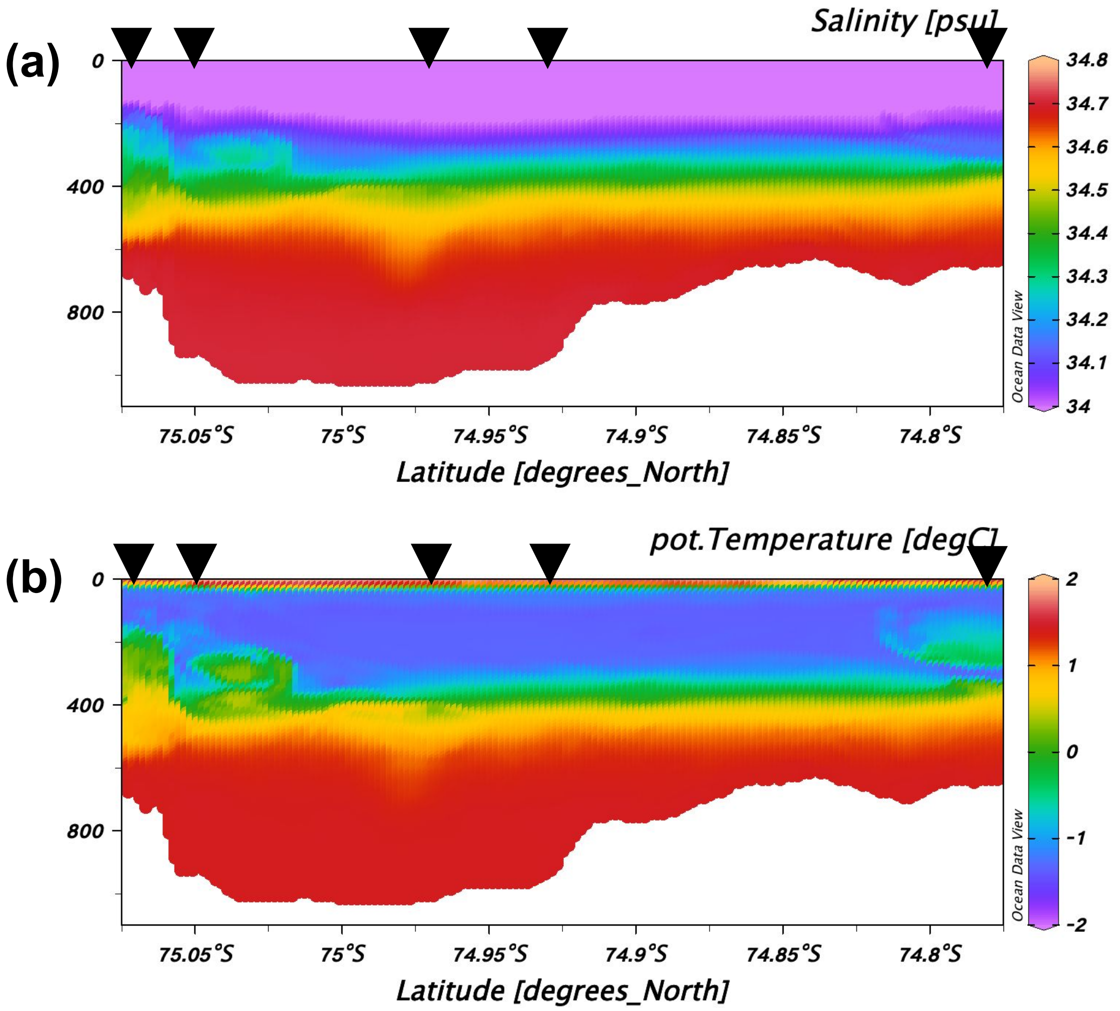


**Supplementary Figure 8.** Simulated vertical sections of (a) salinity and (b) potential temperature on January 30, 2010 along section C in Figure 1. Black triangles indicate the locations of CTD observations.

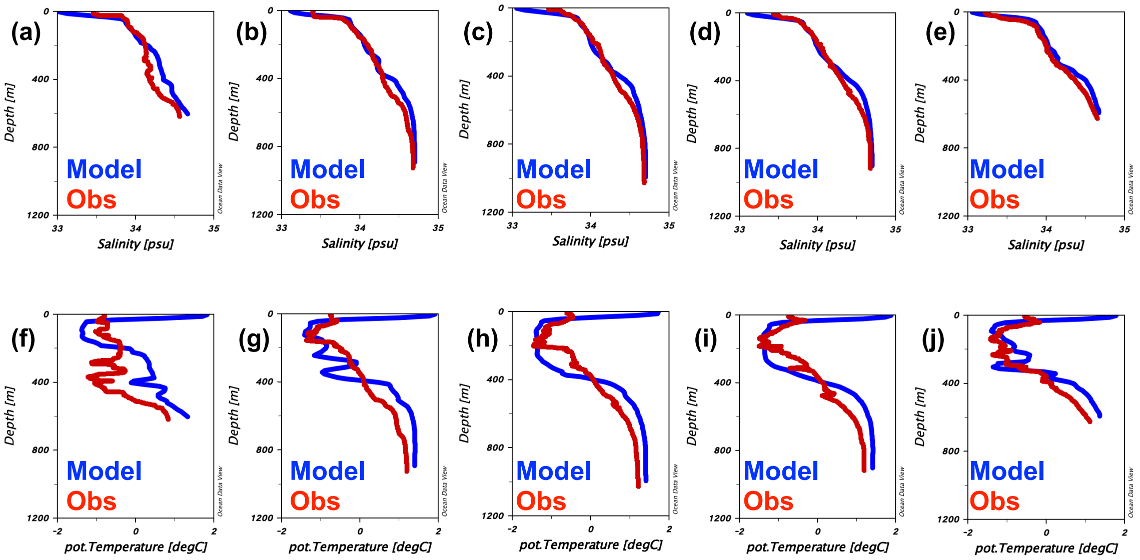


**Supplementary Figure 9.** Observed and simulated vertical profiles of potential temperature and salinity at the locations indicated by the black triangles along section C in Supplementary Figure 8 (ordered from left to right).

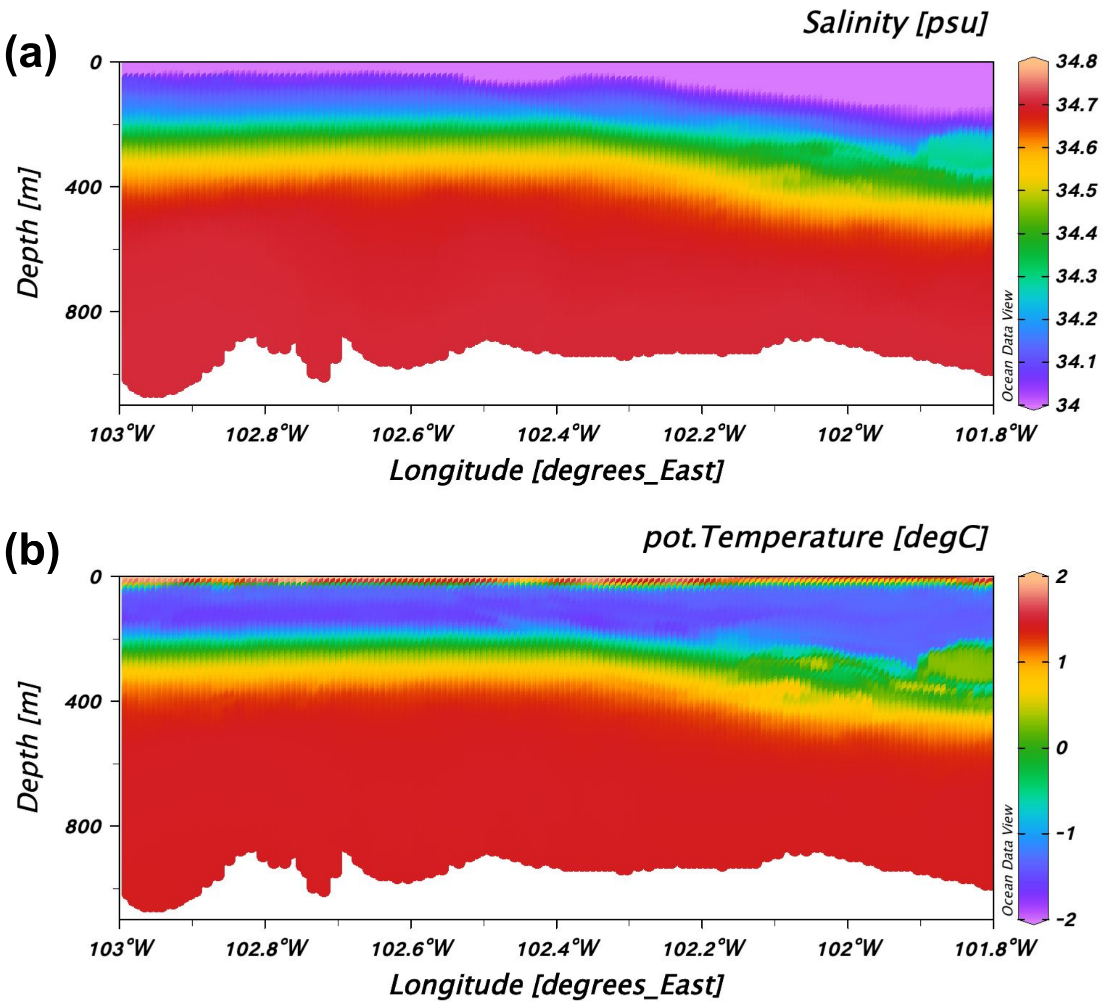


**Supplementary Figure 10.** Simulated vertical sections of (a) salinity and (b) potential temperature on January 30, 2010 along section D in Figure 1.

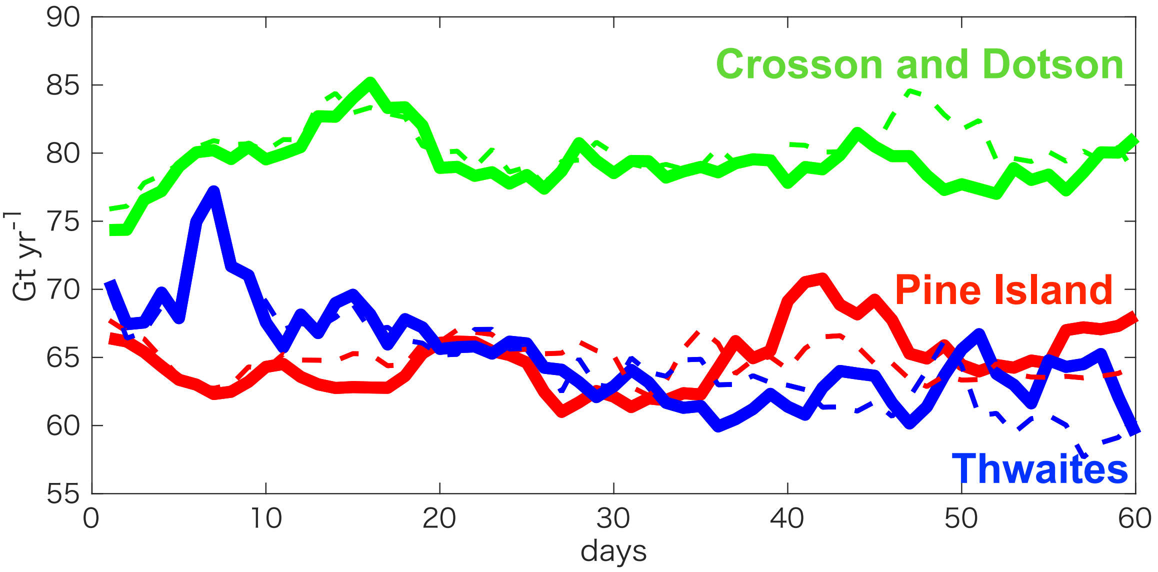


**Supplementary Figure 11.** Simulated time series of basal melt rates from January 1 2010 to March 1 2010 for the Pine Island, Thwaites, and Crosson and Dotson ice shelves for the CTRL (solid) and TIDE (dashed) experiments.

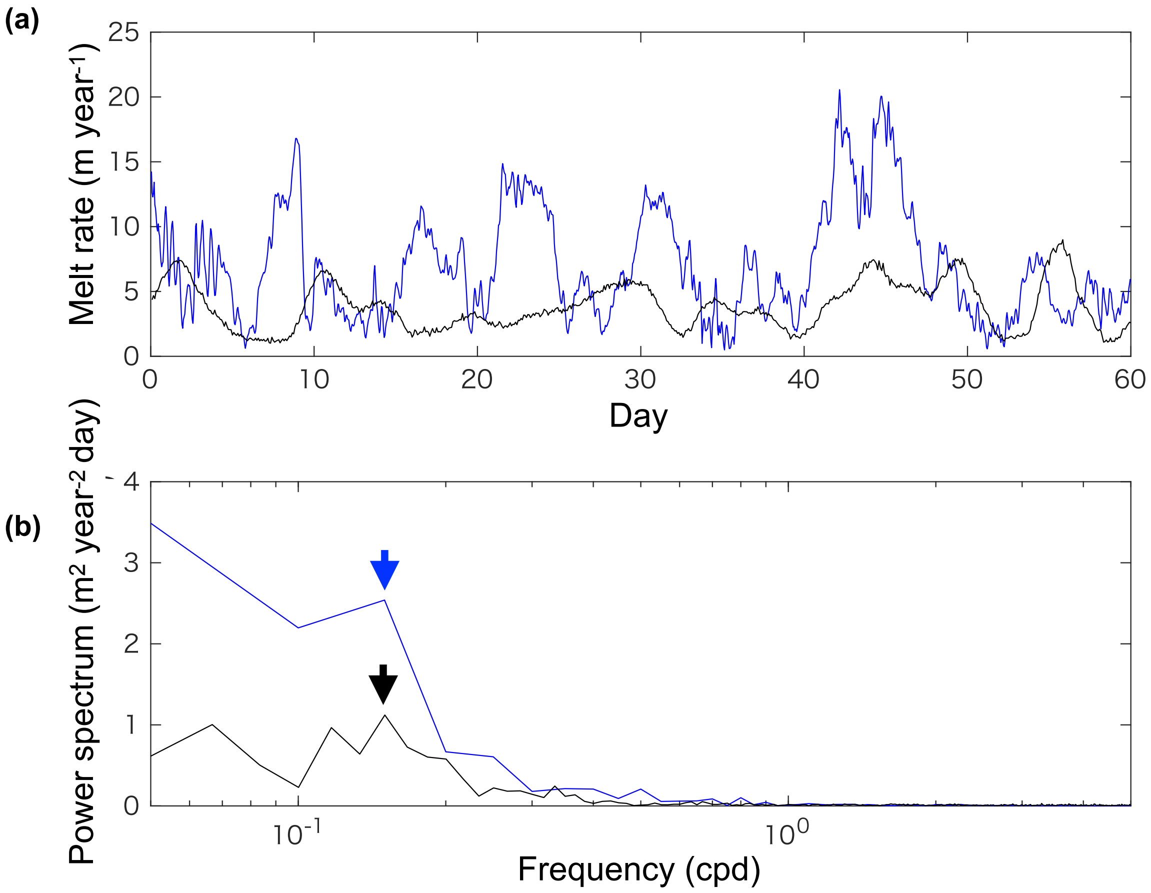


**Supplementary Figure 12.** Time series of observed PIIS melt rate at the location of ApRES measurement (74.97 ºS, 100.99 ºW) (black) and simulated PIIS melt rate at the nearest grid point (101.00ºW, 74.97ºS) for the CTRL case (blue). (b) Power spectrums of observed (black) and simulated (CTRL (blue)) melt rates. Arrows are placed to show the peaks of the spectrums. In-situ ice shelf draft at the location of ApRES observation was approximately 422 m while the prescribed ice shelf draft for our simulation is 492 m. These observations are obtained in 2014 when the thermocline depth was ~200 m deeper[12]. Thus, observed ice shelf melt and ocean speed are expected to be lower and less variable.

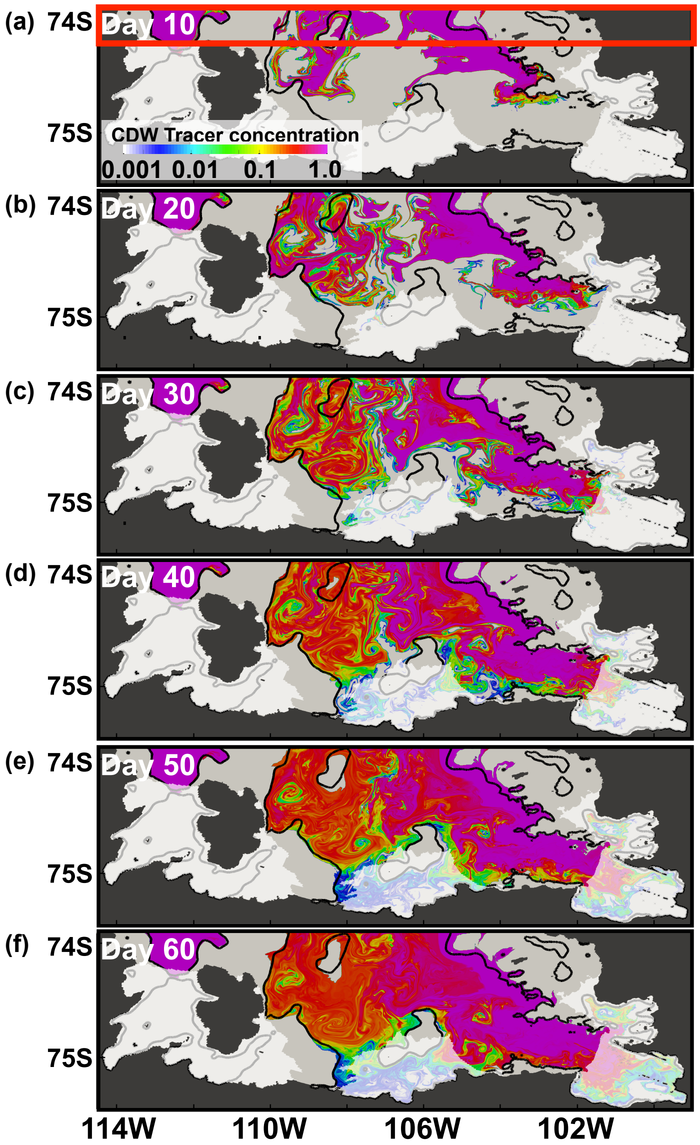


**Supplementary Figure 13.** (a) Spatial distributions of tracers representing CDW after (a) 10, (b) 20, (c) 30, (d) 40, (e) 50, and (f) 60 days of model simulation. The concentration of CDW tracer is set to 1.0 in the region north of 74.24° S (shown by the red box in (a)) and potential temperature higher than 1 ºC. For all panels, bathymetric contours of 500 m are shown (black) and ice shelves are shown with partially-transparent white patches, similar to Figure 1.

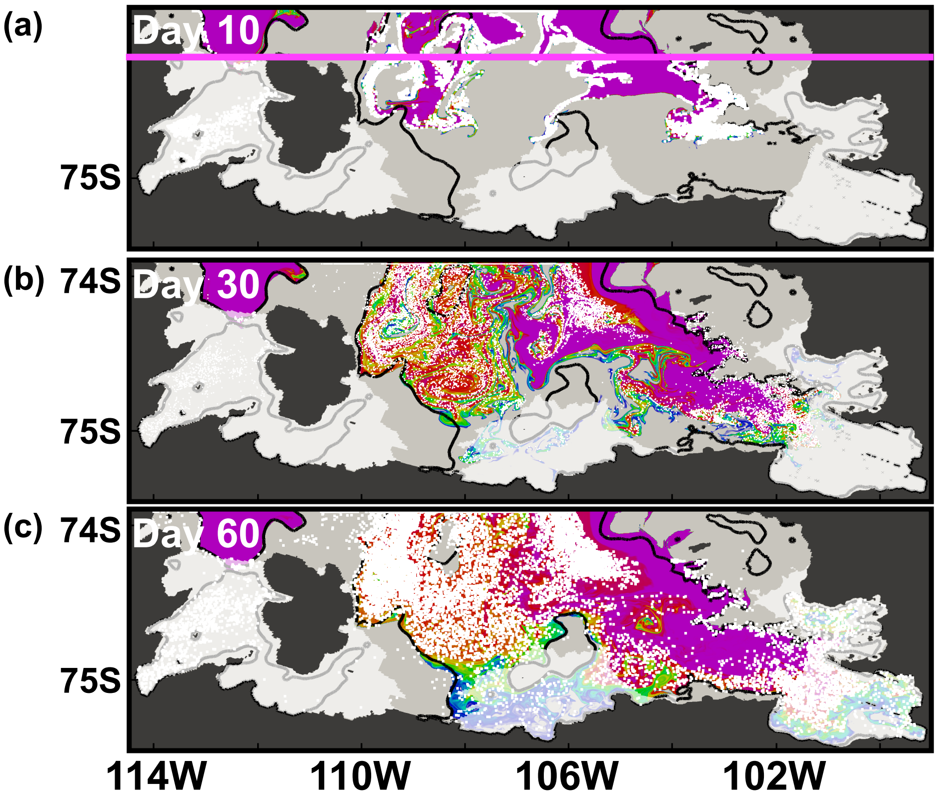


**Supplementary Figure 14.** Spatial distributions of particles (white points) initially released along 74.24º S (pink) after (a) 10, (b) 30, and (c) 60 days of model simulation. Horizontal distributions of CDW tracer (same as Fig. S6) are also shown to indicate that particles and CDW tracer behave similarly. For all panels, bathymetric contours of 500 m are shown (black) and ice shelves are shown with partially-transparent white patches, similar to Figure 1.

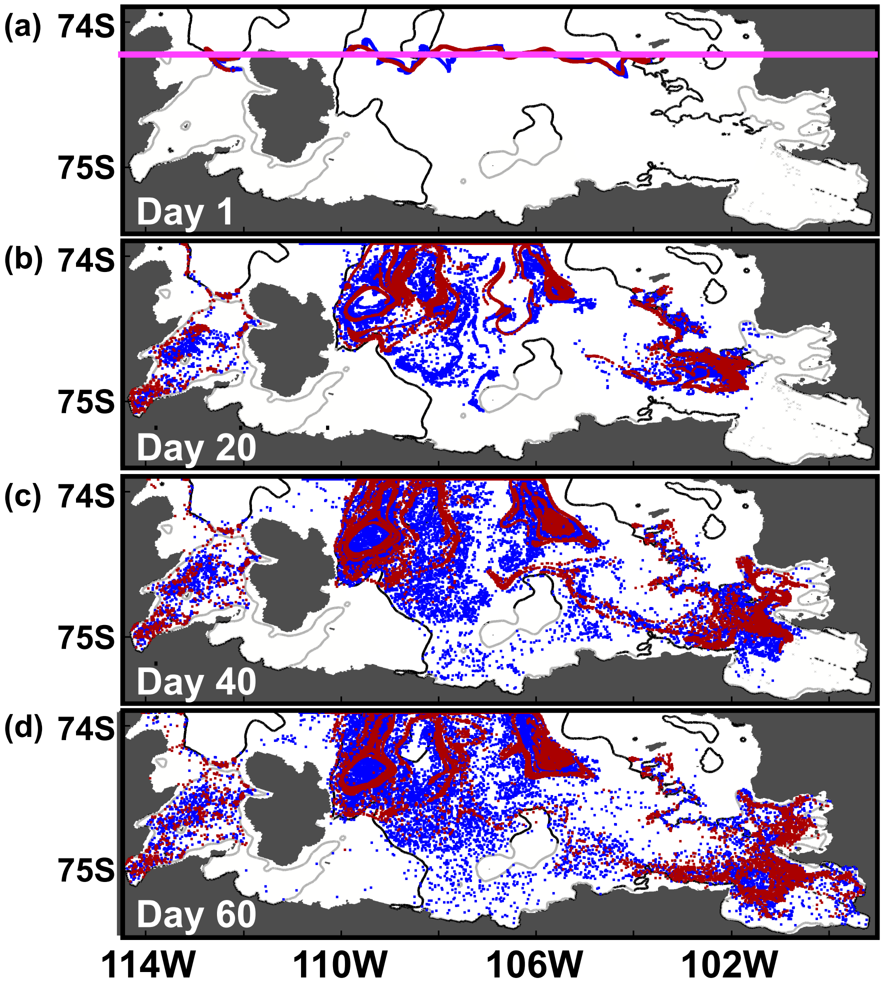


**Supplementary Figure 15.** Spatial distributions of particles initially released along 74.24º S (pink) after (a) 1, (b) 20, (c) 40, and (d) 60 days of model simulation. Red particles change their location based on hourly velocity fields, while blue particles change their location based on the 60-day mean model velocity field. For all panels, bathymetric contours of 500 m are shown (black).

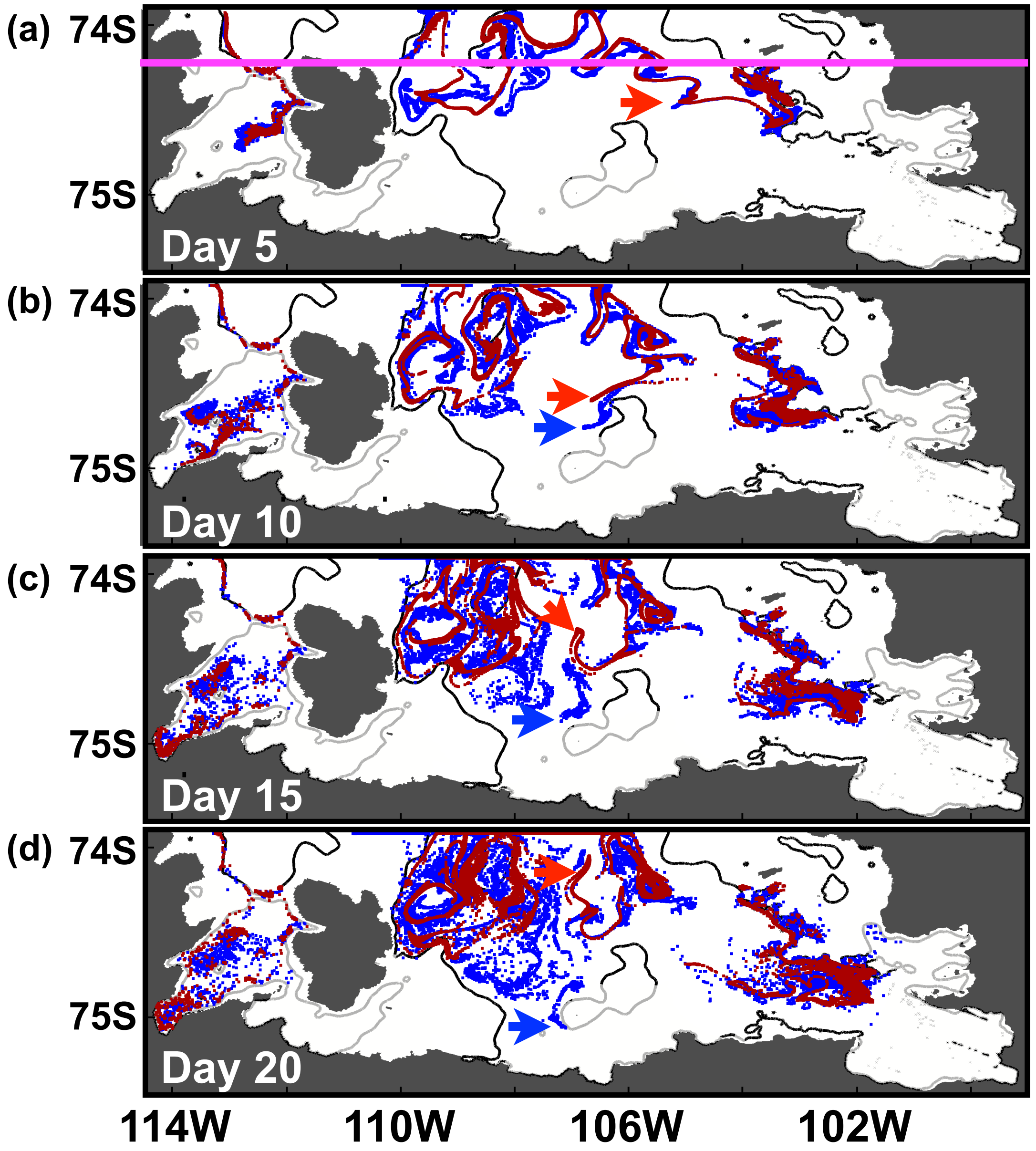


**Supplementary Figure 16.** Same as Fig. S8 but after (a) 5, (b) 10, (c) 15, and (d) 20 days of model simulation. Red and blue arrows are inserted to emphasize the difference of pathways for red and blue particles. After day 20, blue particles (indicated by blue arrow) travel towards the Thwaites ice shelf (TIS) grounding line.

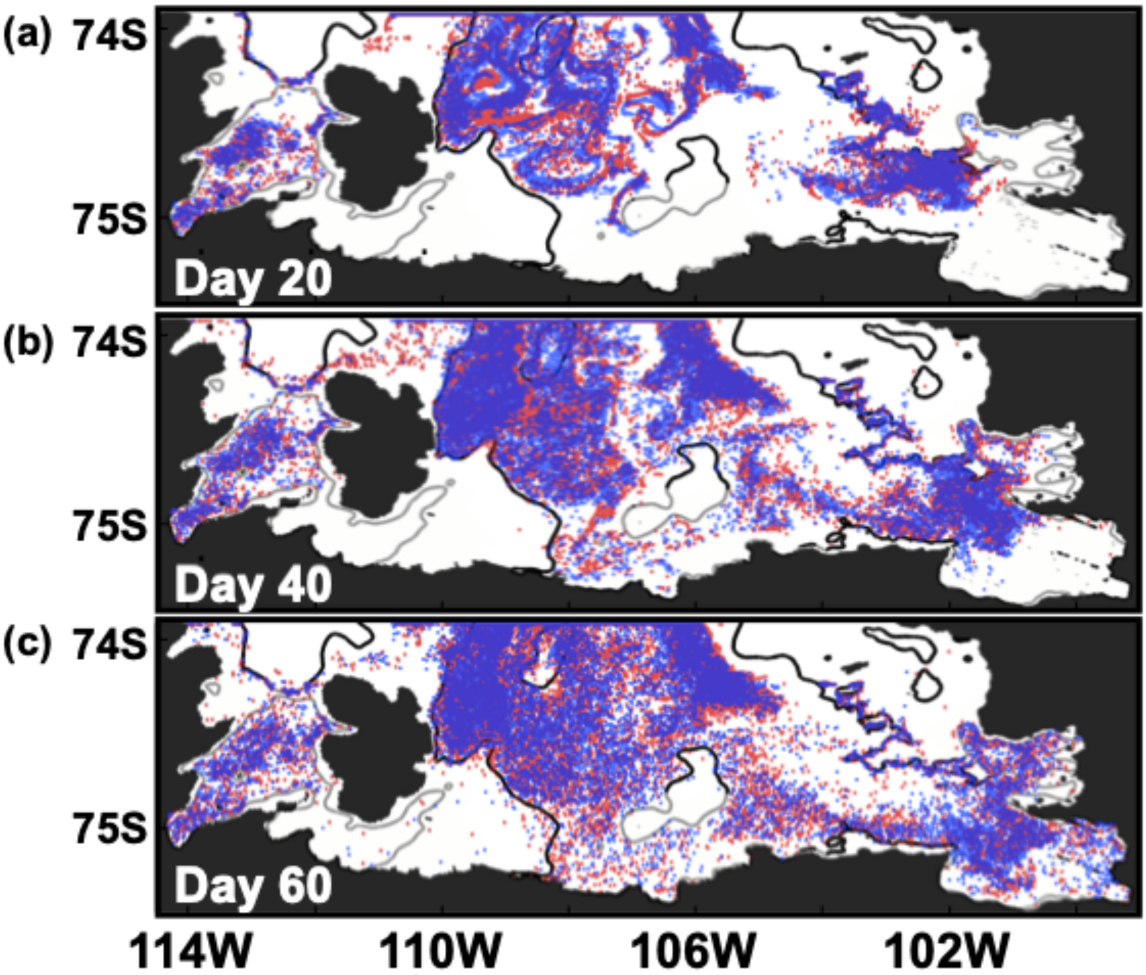


**Supplementary Figure 17**. Spatial distributions of particles initially released along 74.24º S (pink) after (a) 20 (b) 40, and(c) 60 days of model simulation for CTRL (blue) and TIDE (red). For all panels, bathymetric contours of 500 m are shown (black).

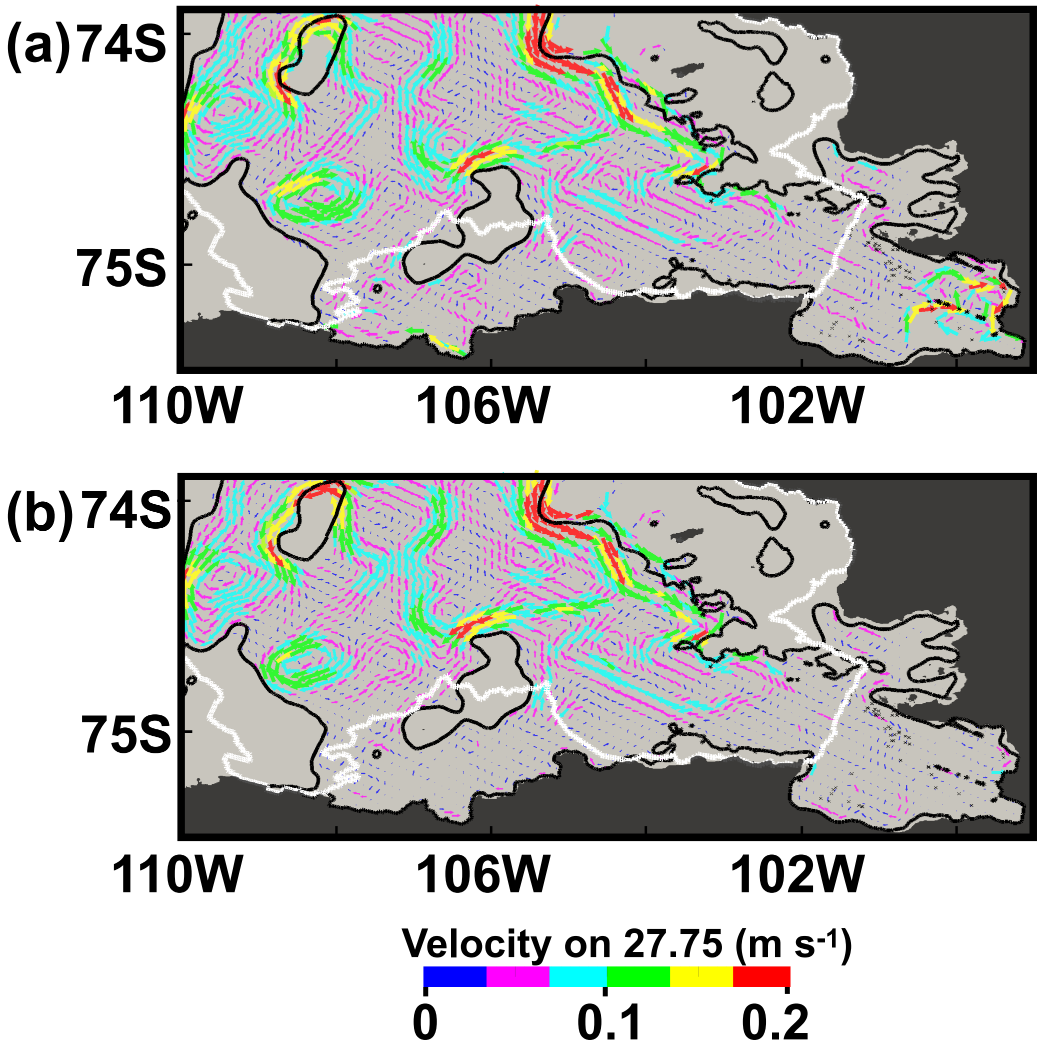


**Supplementary Figure 18.** Horizontal distributions of 60-day mean ocean current along the 27.75 kg m$^{-3}$ isopycnal with directions (arrow) and speed (arrow color) for (a) CTRL and (b) NOMELT. Ice shelf fronts are indicated by the white contours.

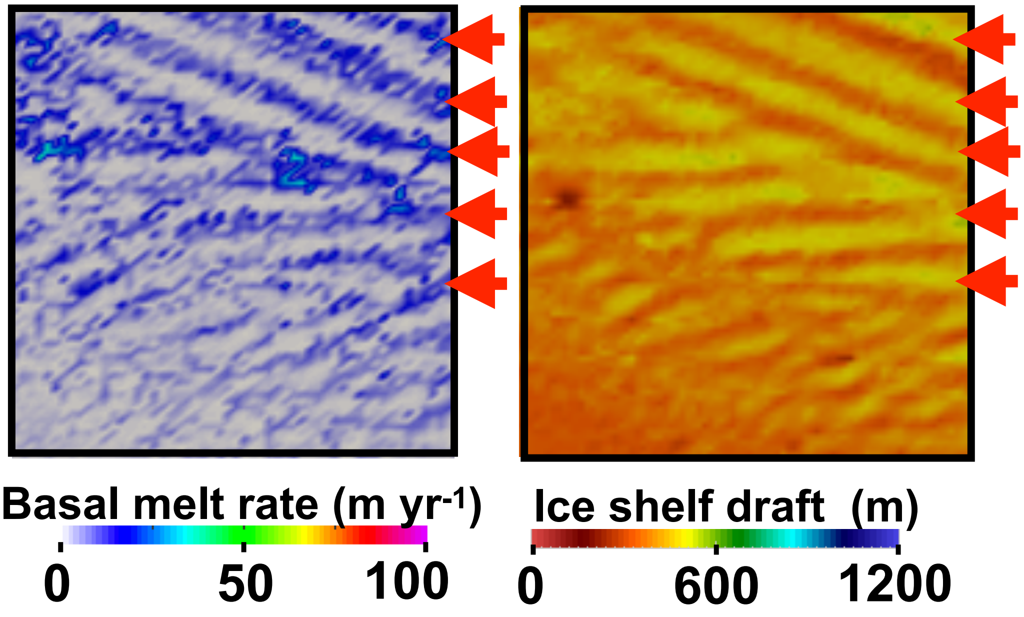


**Supplementary Figure 19.** Close-ups of the PIIS (a) 60-day mean simulated basal melt rate and (b) ice shelf draft for the location enclosed by the black line in Fig. 3b. Red arrows indicate the locations of keels.

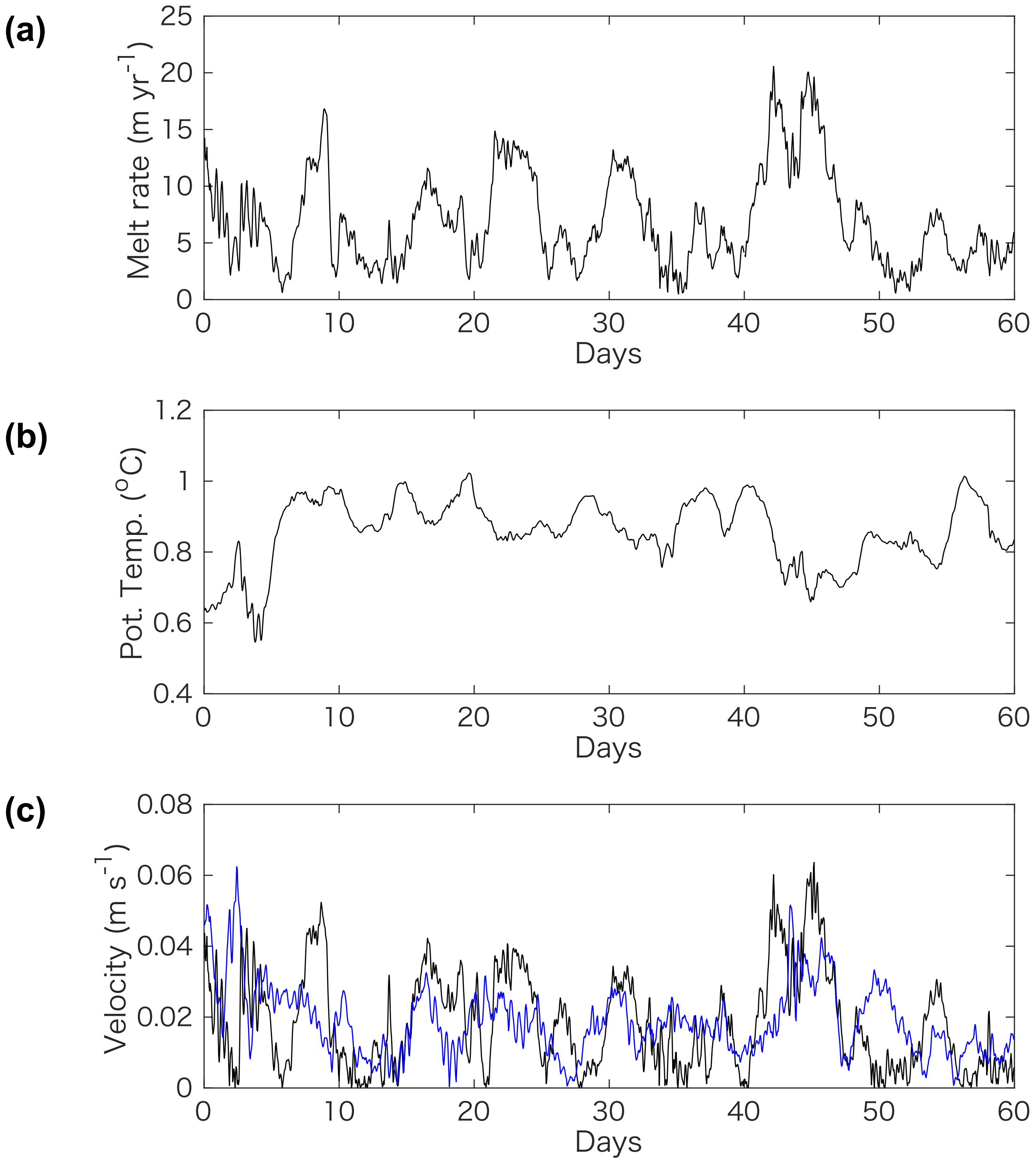


**Supplementary Figure 20.** (a) Time series of (a) simulated PIIS melt rate at the location of ApRES measurements for CTRL. Time series of simulated (b) potential temperature and (c) ocean speed below the ice shelf at the same location. Ocean speed 50 m below the uppermost model grid (blue) is also shown in (c).

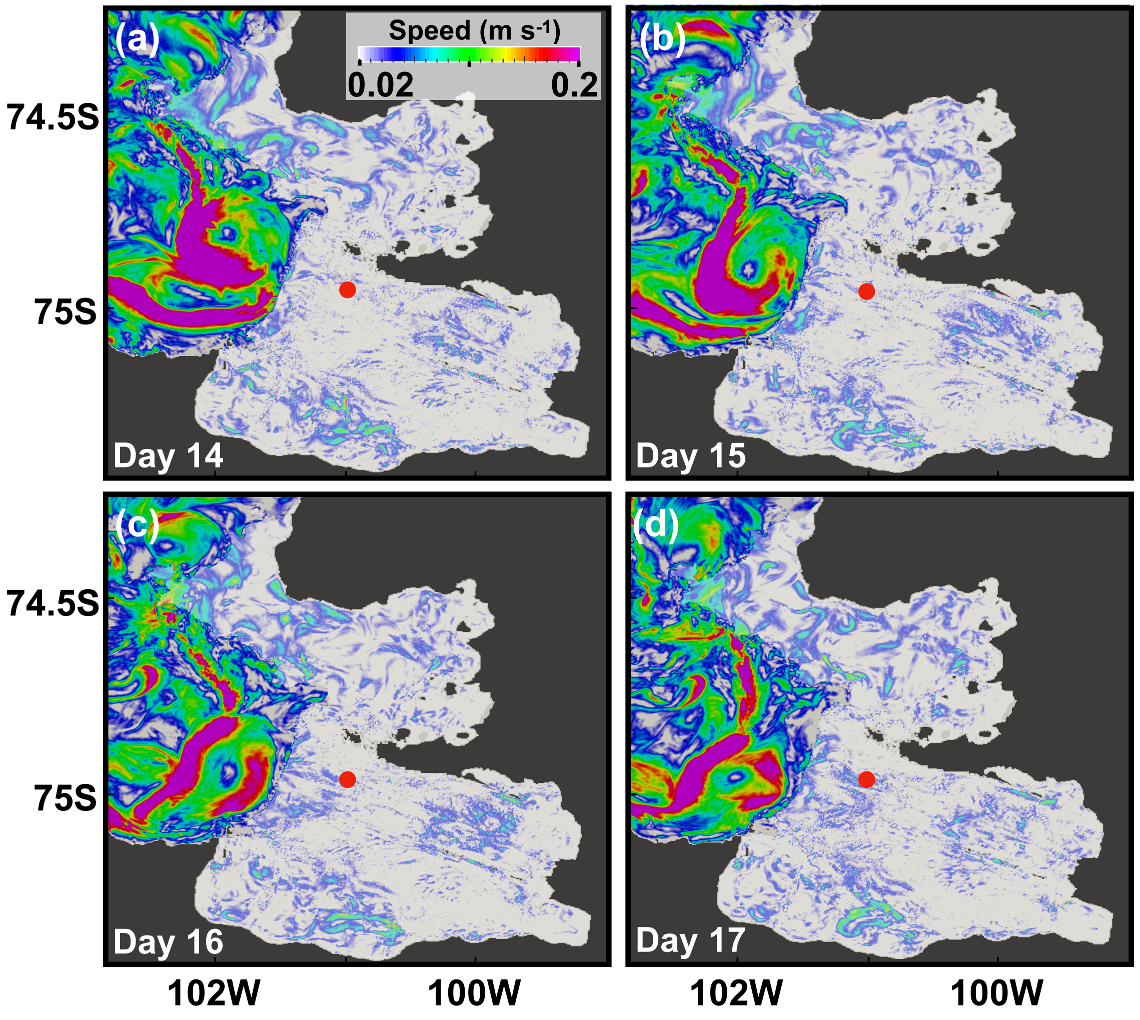


**Supplementary Figure 21.** Ocean speed anomaly 50 m below ocean top cells (below ice shelf for the cavity environment) for days (a) 14, (b) 15, (c) 16, and (d) 17. Ice shelves are shown with partially-transparent white patches, similar to Figure 1.

**Supplementary Table 1.** Model parameters used for simulations in this study. Only parameters that are different from ref. [13] are shown.

| Parameter | |
|---|---|
| Ocean/air drag coefficient scaling factor | 0.25 |
| Air/sea ice drag coefficient | 0.00125 |
| Ocean/air drag coefficient scaling factor | 0.00285 |
| Lead closing (m) | 0.05 |
| Sea ice dry albedo | 0.69 |
| Sea ice wet albedo | 0.61 |
| Snow dry albedo | 0.78 |
| Snow wet albedo | 0.63 |

**Supplementary Table 2.** Description of sensitivity experiments.

| Simulation | Description |
|---|---|
| CTRL | Control simulation |
| TIDE | Tidal forcing is superimposed on lateral ocean boundary forcing |
| NOMELT | Ice shelf melting is prescribed to be zero |
